# Inheritance of Stress Responses via Small Non-Coding RNAs in Invertebrates and Mammals

**DOI:** 10.3390/epigenomes8010001

**Published:** 2023-12-19

**Authors:** Maria C. Ow, Sarah E. Hall

**Affiliations:** 1 Department of Biology, Syracuse University, Syracuse, NY 13210, USA; mcow@syr.edu; 2 Department of Biology and Program in Neuroscience, Syracuse University, Syracuse, NY 13210, USA

**Keywords:** small non-coding RNA, siRNA, miRNA, piRNA, tRF, transgenerational inheritance, intergenerational inheritance, epigenetic

## Abstract

While reports on the generational inheritance of a parental response to stress have been widely reported in animals, the molecular mechanisms behind this phenomenon have only recently emerged. The booming interest in epigenetic inheritance has been facilitated in part by the discovery that small non-coding RNAs are one of its principal conduits. Discovered 30 years ago in the *Caenorhabditis elegans* nematode, these small molecules have since cemented their critical roles in regulating virtually all aspects of eukaryotic development. Here, we provide an overview on the current understanding of epigenetic inheritance in animals, including mice and *C. elegans*, as it pertains to stresses such as temperature, nutritional, and pathogenic encounters. We focus on *C. elegans* to address the mechanistic complexity of how small RNAs target their cohort mRNAs to effect gene expression and how they govern the propagation or termination of generational perdurance in epigenetic inheritance. Presently, while a great amount has been learned regarding the heritability of gene expression states, many more questions remain unanswered and warrant further investigation.

## 1. What Is Epigenetic Inheritance?

In a controversial but influential study in 1942, Conrad Waddington reported changes in *Drosophila melanogaster*’s wing structure due to heat shock stress that persisted for over seven generations despite the lack of the original trigger [1]. This observation was the first account of a phenomenon in which Waddington coined the term “epigenetic”, meaning “above genetics”. Waddington’s well-known “epigenetic landscape” model illustrated how fluctuations in environmental conditions during development can lead to significant changes in phenotype [1,2]. Since then, the definition of the term “epigenetics” has evolved as scientific advances in the field have been made and the molecular mechanisms promoting the epigenetic regulation of gene expression have been identified [3]. The two conditions that spark the least amount of controversy that must be satisfied for a phenomenon to be labeled epigenetic are that stable gene expression changes must not be the result of a modification to the DNA sequence and that these changes are reversible. The discovery of DNA methylation, post-translational histone tail modifications, and non-coding RNAs as the epigenetic mechanisms that regulate gene expression both spatiotemporally during development and as a result of environmental factors satisfies these first two conditions [4]. The third requirement for a gene expression change to be labeled epigenetic is that it must be heritable. However, the definition of what is considered “heritable” has evolved during the modern era of molecular genetics [5]. Originally, the heritable component was only valid when gene expression states were inherited across generations, but a more modern, inclusive definition is that heritability can refer to the maintenance of gene expression states across cell divisions, or even a prolonged change in gene expression for post-mitotic cells [4]. For the purposes of this review, we will focus on the mechanisms of epigenetic inheritance across one or more generations.

**Figure 1:**
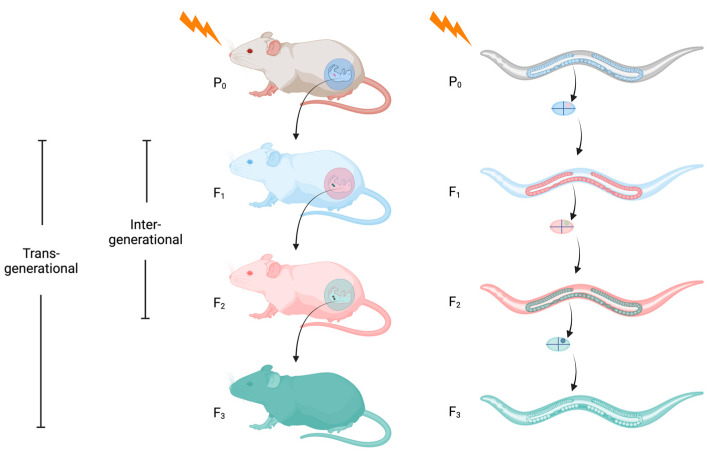
Intergenerational versus transgenerational inheritance. In mice and *C. elegans*, transgenerational inheritance first occurs in the F_3_ generation. Colors indicate cells derived from the same population.

Intergenerational epigenetic inheritance or parental inheritance refers to the passing on of a parental (P_0_) trait to the next generation (F_1_), such as maternal provisioning via the placenta in mammals. For example, a P_0_ gestating mother who is directly exposed to an environmental factor is not only exposed herself but also exposes her F_1_ generation progeny and their developing germ cells that will produce the F_2_ generation. Thus, in mammals, the F_3_ generation is the first generation that was not exposed to the original environmental factor (Figure 1). Transgenerational epigenetic inheritance (TEI) is the durable epigenetic inheritance that persists beyond parental inheritance, and requires at least two generations for paternal transmission and at least three generations for maternal transmission in animals in which embryonic development occurs in utero [6]. In other animals, such as *Caenorhabditis elegans* nematodes, the P_0_ hermaphrodite, its germ cells, and any unhatched F_1_ embryos still in the uterus are exposed to the environmental factor. The germline lineage in the F_1_ progeny is specified at the four-cell embryonic stage, before the egg has been laid [7]. Thus, similar to mammals, the F_3_ generation is the first generation of *C. elegans* not exposed to the original environmental factor (Figure 1). Epigenetic inheritance is contingent on germ cells carrying an inherited “factor”, which then proceeds to transform the epigenome of the descendant embryo to promote phenotypic changes [8,9,10,11].

Signs of potential epigenetic inheritance in humans can be found throughout recorded history, where natural disasters, human-made or otherwise, have taken devastating tolls on human populations. For example, two of the most notorious recent human famines include the Dutch Winter Hunger of World War II (1944–1945) and the Great Chinese Famine (1958–1962), the latter of which is widely regarded as the deadliest famine in human history with ~30 million deaths [12,13]. Individuals that survived in utero exposure to these famines were more likely to exhibit metabolic disease in adulthood compared to unexposed siblings [14]. In the case of the Dutch Hunger Winter, metabolic disease in the affected individuals is correlated with changes in DNA methylation levels at cytosine-guanine dinucleotides (CpGs) associated with genes that have functions in insulin signaling and metabolism [15]. Simulations that model the correlations between famine exposure, cytosine methylation levels, and metabolic disease suggest that selection, and not plasticity, may play a role in shaping the epigenome of famine survivors. Fetuses with a favorable set of epialleles resulting from the stochastic re-methylation of the genome during development may have had an increased rate of survival in adverse uterine conditions [16]. However, generational studies have found that the descendants of famine survivors are also disproportionately more vulnerable to disease [14,17,18]. Grandchildren of survivors from the Dutch famine were more likely to experience obesity, cardiovascular disease, and metabolic disorders, and the incidence of pulmonary tuberculosis increases for two generations following prenatal or early-life exposure to the Great Chinese Famine [18]. Whether the descendants of famine survivors exhibit metabolic disease due to the inheritance of specific DNA methylation patterns established in the affected ancestor remains to be determined. In addition, children of parents who experienced trauma-inducing events, such as the Holocaust, show increased vulnerability to physical, behavioral, and neurological disorders [19]. More recently, pregnant women that were significantly impacted by Hurricane Sandy on the northeast coast of the United States in 2012 now have children with a higher incidence of anxiety and aggression [20]. Each distinct, early-life stress results in different adult phenotypes, presumably that allow the affected individual and their future generations to better adapt to that particular environmental stress experience. However, disease can occur when the adaptive phenotype and the current environment are mismatched [21].

Epigenetic inheritance due to the environmental experiences of the parental generation is not limited to humans and is well documented in the animal kingdom. Elevating glucocorticoid hormones in meerkat pregnant females dampens the growth of their female offspring while also increasing the offspring’s pup rearing and feeding behaviors [22]. Increased dietary sugar in *Drosophila* fathers reprograms the chromatin state and elicits obesity in their offspring [23]. Maternal malnutrition in the crustacean *Daphnia magna* results in the production of progeny that are slow-feeders [24]. Juvenile wild baboons whose mothers experienced adversity (being orphaned before reaching adulthood) were significantly more likely to die at a younger age than those whose mothers were not orphaned [25]. Abiotic (CO_2_ level, pollutants, salinity level) and biotic (viruses, predators) environmental factors affect offspring phenotypes (metabolism, shell structure, growth, morphology, lifespan) in various mollusk taxa [26]. In zebrafish, paternal exposure to the common herbicide atrazine changes their offspring’s behavioral traits and causes serotonergic system dysregulation [27]. Parental contact with aquaculture plastic contaminants altered the swimming behavior, development, and growth of Pacific oyster progeny [28]. Finally, parental early-life starvation in turnip sawflies affects the life history and consumption traits of their offspring larvae [29]. While this is not an exhaustive list of examples, the common theme is that parental environmental experience triggers the inheritance of an environmental “memory”, leading to altered phenotypes in the progeny.

The above examples in humans and other animals are intriguing, but we are left to wonder whether these observations are labeled “epigenetic” for lack of a better explanation. Despite an explosion of research investigating the mechanisms of epigenetic phenomena in the last few decades, we still understand very little about the mechanism of how different environmental experiences can target specific gene expression changes in subsequent generations. While there is clearly enough evidence of epigenetic inheritance, one of the challenges is a concept known as the Weismann barrier, which states that germ cells and somatic cells are separate and genetic information flows only from germ cells to soma, not vice versa [30]. While clearly there is enough evidence to disprove this model now, the mechanism of how sensory experiences of somatic tissue can reprogram germ cells to retain a memory of environmental experience is not well understood. In this review, we will highlight the most recent examples demonstrating how animals use small non-coding RNAs (sncRNAs) to promote epigenetic inheritance. We also address recent advances in the mechanisms of inheritance, such as how genes become targeted by sncRNAs to propagate silencing across generations.

## 2. Biogenesis and Functions of sncRNAs

One of the unanswered questions of epigenetic inheritance is how a “memory” is inherited via germ cells. Two constituents of epigenetic inheritance are histone modifications and DNA methylation, but they are more cumbersome to establish or inherit, ostensibly due to zygote resetting to ensure totipotency [31,32,33], while sncRNAs lend themselves as more flexible for inheritance and propagation. Nevertheless, the roles of histone and inheritance in living organisms is well chronicled, and the reader is invited to consider several excellent reviews that cover the topic extensively [34,35,36,37,38,39,40]. DNA methylation is an epigenetic footprint and the topic of its heritability has been covered elsewhere [41,42,43]. The biogenesis of small interfering RNAs (siRNAs), Piwi-interacting RNAs (piRNAs), and microRNAs (miRNAs) has been discussed in detail elsewhere (see [44]); thus, here, we present only a brief overview of the biogenesis and functions of different small RNA classes that have been implicated in epigenetic inheritance.

### 2.1. Small Interfering RNAs

This class of siRNAs can be subdivided into two types that differ in the source of the RNAi trigger: exogenous siRNAs (exo-siRNAs) and endogenous siRNAs (endo-siRNA). RNA interference (RNAi) triggered by an externally provided source, such as feeding worms with bacteria expressing double-stranded RNA (dsRNA), injection into the gonads, or soaking in dsRNA, is referred to as exo-RNAi or commonly referred to as RNAi [45]. On the other hand, the production of endo-siRNAs is stimulated by environmental factors [46], such as biotic or abiotic stresses.

Epigenetic inheritance was one of the first characteristics described of RNAi [47,48]. The development of negative-strand nucleic acid as a laboratory tool to inhibit gene expression was based on reports that naturally occurring anti-sense nucleic acids interfere with cellular processes, including ribosome function, plasmid replication, and RNA splicing [49]. An RNAi-type phenomenon was reported in the petunia plant when researchers manipulating the anthocyanin biosynthesis pathway, responsible for its vibrant color, inadvertently generated white petunias [47]. A similar phenomenon that caused a “quelling” effect of an endogenous gene due to the introduction of homologous sequences was reported a couple of years later in *Neurospora crassa* [50]. Similar gene silencing outcomes in an animal, using either sense or anti-sense RNAs, were first documented in the *C. elegans* nematode [48,51]. It was not until several years later that Fire and colleagues reported in their seminal study that dsRNA, rather than individual strands, results in robust mRNA-specific silencing that propagates between tissues, indiscriminate of the soma and germline boundaries, and is heritable by naïve progeny. Because only a few dsRNA molecules were necessary for this RNA interference phenomenon, a propagatable mechanism for the intercellular and intergenerational trafficking of silencing factors that can escape the degradation of RNA transcripts during embryogenesis was postulated to exist [52]. Indeed, the introduction of dsRNA into one tissue in a parental worm, its spread to other tissues, and its inheritance by the progeny depended on the components of systemic RNAi, including the conserved dsRNA channel, SID-1 [53,54,55].

In some species, such as *Drosophila* and *C. elegans*, dsRNA is the trigger for RNAi processes to be initiated and propagated across generations [56,57,58]. DsRNA is recognized by the RNase III ribonuclease, Dicer, which cleaves it into siRNAs ranging between 20 and 30 nucleotides depending on the species [59,60]. The siRNAs bind to an Argonaute (AGO), which acts as an effector protein to silence gene expression via mRNA cleavage in the cytoplasm (post-transcriptional gene silencing) or by targeting nascent RNAs in the nucleus and recruiting heterochromatin formation complexes to the gene locus (transcriptional gene silencing) [61,62]. SiRNAs direct AGOs to an RNA target by binding with perfect anti-sense complementarity [46,63,64,65]. In order for gene silencing to be inherited, the siRNAs must be propagated so that they do not dilute in concentration after many cell divisions. In *C. elegans* and plants, new dsRNAs for Dicer processing are produced by RNA-dependent RNA polymerases (RdRPs) generating an anti-sense copy of an mRNA. However, no RdRP or similar enzyme has been identified to date in *Drosophila* species or mammals, so the propagation of siRNAs as a mechanism of epigenetic inheritance in these animals is unclear [66].

In *C. elegans*, the biogenesis of siRNAs includes an extra step due to the addition of an extra category of siRNAs. Primary siRNAs are 26 nucleotides long and are enriched for a guanine at their 5’ end (26G siRNAs). This class of siRNA is generated via the Dicer-dependent cleavage of dsRNAs and is found in relative low abundance. The second class of siRNA is Dicer-independent and made by RdRPs making anti-sense RNAs that are 22 nucleotides long and are enriched for a 5’ guanine (22G siRNAs). *C. elegans* has 19 functional AGOs, which have specificity based on the type of small RNA they bind, the tissue types where they are expressed, and whether they act via transcriptional or post-transcriptional gene regulation [63]. CSR-1 is the only essential AGO, and it functions to protect “self” gene expression by promoting a favorable chromatin environment for transcription [67,68,69,70]. Acting in opposition to CSR-1, the worm-specific Argonautes, or WAGOs, act to silence “non-self” components of the genome. One WAGO, the germline-expressed nuclear HRDE-1 (Heritable RNAi Deficient 1)/WAGO-9, is the principal Argonaute in the maintenance of transgenerational inheritance. HRDE-1 is dispensable for the initial induction of this heritable process and shuttles between the cytoplasm (the loading site of small RNAs) and the nucleus (the action site) and physically associates with 22Gs siRNAs [71]. The inheritance of an initiated silence state via HRDE-1 is dependent on the continued amplification of 22G siRNAs by RdRPs, RRF-1, and EGO-1, and typically lasts between three and five generations [71,72,73,74].

### 2.2. Piwi-Interacting RNAs

Mobile genetic elements such as transposable elements (TEs) are present in almost all prokaryotic and eukaryotic genomes. TEs can constitute about 16% of *C. elegans*, 20% of *Drosophila*, 37% of mouse, 44% of human, and up to 85% of some plant genomes [75,76,77,78,79]. While selfish genetic elements are major contributors to genetic diversification and evolution [79,80,81], their ubiquitous parasitic presence poses a unique challenge to their host’s effort to preserve the integrity of its genome and the perdurance of its species. A number of human pathologies, including hemophilia, X-linked dystonia–parkinsonism, autoimmune disorders, and certain cancers, have been linked to the uncontrolled transposition of mobile genetic elements [82,83]. Some human pathological conditions, such as cancers caused by the loss of the p53 tumor suppressor activity in repressing rampant transposon activity, have been proposed to fall under a novel class of diseases called “transposopathies” [84,85,86]. 

Animals have devised a transposable element silencing strategy by which Piwi (p-element induced wimpy testis)-clade AGO proteins and their associated 23-32-nucleotide-long piRNAs, or piRISC complexes, function in the germline and the soma to detect and silence complementary RNA targets [87,88,89]. The earliest indication of the function of piRNAs in heritable silencing in the fruit fly *Drosophila melanogaster* was exhibited via hybrid dysgenesis, a genome incompatibility phenomenon whereby the progeny of crosses between different *Drosophila* strains may be sterile due to transposon activation [90,91,92]. Evidence for the piRNA pathway in transgenerational epigenetic silencing has also emerged in *C. elegans* where the PRG-1 Piwi protein can initiate, but not maintain, stable silencing of a transgene or non-self RNA for multiple generations. In these cases, the maintenance of transgenerational epigenetic silencing instead depends on RNAi and chromatin modifiers [73,93,94,95,96].

*Drosophila* piRNAs originate from large piRNA gene clusters located in the pericentromeric and sub-telomeric regions that can be several thousand base pairs long. These piRNAs clusters are rich in repetitive sequences, mostly relics of transposable elements, and are transcribed as long precursors that are processed into mature piRNAs [97,98]. Transcription of piRNA clusters is conducted by a non-canonical RNA II polymerase and cluster-specific transcription accessory proteins governed by chromatin marks. Most piRNA clusters are transcribed from both strands (dual-strand clusters) and are the primary source of *Drosophila* germ cell piRNAs [88,97,99,100,101]. Although the piRNA clusters in the euchromatic–pericentromeric borders producing most the piRNAs in the *Drosophila* gonads have been considered the main regulators of transposon activity [88,97,102], a recent study presented evidence of their transitory nature and expendable role in transposon defense and fertility at an evolutionary scale [103].

### 2.3. MicroRNAs

First reported 30 years ago in the nematode *C. elegans*, microRNAs (miRNAs) are the founding class of sncRNAs. They were found not just to be peculiarities of a nematode genome, but were highly conserved in other animals, including humans [104,105]. Since then, miRNAs have been identified in nearly all eukaryotic genomes from plants to humans, and their function encompasses virtually all aspects of organismal biology, including human pathologies [106,107,108,109,110,111]. The mature miRNA sequences range in number from >400 in *Arabidopsis thaliana*, *C. elegans*, and *D. melanogaster* to >2000 in humans [112]. The biogenesis of a miRNA begins with transcription from a genomic miRNA gene by RNA polymerase II. The resulting hairpin-structured primary miRNA (pri-miRNA) is processed by a microprocessor complex consisting of the RNase III enzyme, Drosha, and Pasha (DGCR8) to yield a shorter double-stranded hairpin structure precursor miRNA (pre-miRNA), which is exported to the cytoplasm by the Exportin5 and RAN-GTP complex. Once in the cytoplasm, the pre-miRNA undergoes further processing by Dicer to produce a ~22-nucleotide-long mature miRNA. MiRNAs complex with AGOs in miRISC silencing complexes to effect gene expression changes in a post-transcriptional or translational-repressive manner via imperfect base-pairing to the 3’ untranslated region (3’UTR) of the target mRNAs [107,113,114,115].

A necessary mechanism for epigenetic inheritance is the ability to amplify the small RNA silencing agents in order to evade dilution in subsequent generations in the absence of the original trigger, as is the case for piRNAs and siRNAs [72,88,116]. Since miRNAs lack an amplification mechanism, the role of miRNAs in epigenetic inheritance is not clear. However, studies in mice have suggested that miRNAs play a role in the inheritance of a phenotypic trait by the offspring (i.e., intergenerational epigenetic inheritance) [117,118,119,120,121,122]. To this effect, the biogenesis of siRNAs in *C. elegans* has been reported to be orchestrated by a miRNA [123], thus leaving open the mechanistic prospect that miRNAs can affect the transgenerational transmission of an ancestral trait.

### 2.4. Transfer RNA-Derived Fragments

Transfer RNAs (tRNAs) are non-coding RNAs that bridge the processes of transcription and translation. Their anticodon loops base-pair with mRNA codons in the ribosome to deliver amino acids for polypeptide formation. While fragments of tRNAs were first observed in the urine of cancer patients and in *Escherichia coli* following bacteriophage T4 infection [124,125], these transfer RNA-derived fragments (tRFs) were later revealed to be not just debris from tRNA degradation or biogenesis by-products but instead result from non-random tRNA processing events that are required for cell proliferation [126]. Also referred to as tsRNAs (tRNA-derived small RNAs) or tiRNAs (stress-induced tsRNAs) [127,128,129,130], several classes of tRFs (5′-tRNA half, 3′-tRNA half, tRF-1, 5′U-tRF, 3′-tRF, 5′-tRF, and i-tRF), ranging in size from ~13 to 48 nucleotides, have been classified based on where they map on their pre-tRNA or mature tRNA precursors [131,132,133]. Following the transcription of tRNA genes in the genome by RNA polymerase III [134], the maturation of pre-tRNAs starts with the removal of the 5’ leader and 3’-poly U by endoribonuclease RNase P and ribonuclease RNase Z (ELAC2), respectively, and the addition of a 3’ CCA to the 3’ acceptor stem by tRNA nucleotidyltransferase [135,136,137,138]. It is during this tRNA maturation process that a diverse class of tRFs is generated from the combinatorial action of a diverse group of known and unknown endoribonucleases and exoribonucleases, including RNase T2, ANG, RNase L, SFLN3 (RNase S13), and RNase Z (ELAC2), and tRNA modification enzymes, such as methyltransferases BCDIN3D, TRMT10A, NSUN2, DNMT2, pseudouridine synthase PUS7, and tRNA demethylase ALKBH3 [139].

tRFs have been identified in all domains of life, and an appreciation for their critical roles in gene regulation and human health and disease has boomed in the past decade [139,140,141,142,143,144]. Extracellular vesicles (EVs) secreted by most cells that function in cell-to-cell communication and host–pathogen interactions [145,146] were found to be enriched with tRFs, suggesting the important role of tRFs in the dissemination of signals between tissues and with the environment of an organism [133,147,148]. In addition, tRFs are promising biomarkers and therapeutics for human disease [133,149,150,151], and function in the regulation of gene expression [127,148,152,153], stress or immune responses [128,147,154,155,156], and intergenerational epigenetic inheritance via sperm [127,129,130,157,158].

### 2.5. Small Nucleolar RNAs 

Small nucleolar RNAs (snoRNAs) are an ancient class of short non-coding RNAs present in plants and animals. While their integral roles as modifiers of RNA (e.g., ribose 2′O-methylation and pseudouridylation of rRNAs) and as components of the spliceosome are well established, their possible functions in post-transcriptional regulation have begun to emerge. Similar to tRFs derived from tRNA processing, snoRNA-derived RNAs (sdRNAs) are generated from the cleavage of different types of mature snoRNAs. One type of sdRNA fragment has been shown to associate with AGO1 and AGO2 in human embryonic kidney cells and target an endogenous mRNA, suggesting that some sdRNAs may function similarly to miRNAs [159,160]. SdRNAs have also been associated with PIWI proteins and regulate gene expression via mRNA decay and the recruitment of histone modification complexes [161,162]. While sdRNAs have not yet been demonstrated to promote epigenetic inheritance across generations, their ability to regulate gene expression with miRNA and piRNA-like mechanisms suggests their repertoire of functions may expand in the future to include emissaries of epigenetic inheritance [163,164,165,166]. 

## 3. Germ Granules and Epigenetic Inheritance

Germ granules, also referred to as nuage, are germline-specific membrane-less perinuclear assemblages of RNA-binding proteins and RNAs located outside the nucleus that have been identified in numerous species throughout the animal kingdom. They are important for germ cell development, mRNA translation, and RNA metabolism. It is estimated that most mRNAs depart to the cytoplasm from the nucleus via these germ granules [167,168,169]. In *C. elegans*, germ granules consist of a collection of at least four neighboring, yet separate, condensates with distinguishable roles: P granules, Z granules, Mutator foci, and SIMR foci. It is in these compartments that certain proteins involved in the sncRNA pathways localize [170,171,172]. 

The largest and best characterized germ granule component is the P granule, where numerous proteins localize, including PGL-1/-3, Vasa-DEAD like helicases GLH-1/-2/-3/-4, RdRP EGO-1, and six AGOs (ALG-3/-4/-5, PRG-1, CSR-1, and WAGO-1). The P granule is hypothesized to be where *C. elegans* RNA surveillance or recognition takes place [67,72,173,174,175,176,177,178,179]. Z granules are enriched for the RNA helicase ZNFX-1, piRNA biogenesis factor PID-2/ZSP1, and AGO WAGO-4. Increasing evidence suggests that Z granules are vital for RNAi inheritance [169,170,180,181,182,183]. SncRNA amplification is hypothesized to occur in the Mutator foci, where the proteins required for siRNA biogenesis localize, including endoribonuclease RDE-8, RdRP RRF-1, the Mutator proteins MUT-2/RDE-3, MUT-7, MUT-8/RDE-2, MUT-14, and MUT-15, and the Mutator scaffold protein MUT-16 [169,172,184,185,186,187]. The core protein in the SIMR focus is the Tudor domain protein SIMR-1, which functions downstream of PRG-1-dependent biogenesis but upstream of the Mutator complex siRNA amplification complex [171]. Also residing in the SIMR foci is the RSD-2 protein, which is required for exogenous RNAi in *C. elegans* [171].

Recent studies have revealed the important roles of germ granules in *C. elegans* epigenetic inheritance, particularly the Z granules. In an effort to identify genes important to epigenetic inheritance, two groups contemporaneously performed genetic screens and identified mutations in *znfx-1*/*zk1067.2* and *wago-4* as disrupting RNAi inheritance [170,180]. *Znfx-1* is predicted to encode a protein with a superfamily one (SF1) RNA helicase domain and a zinc-finger domain with orthologs in most eukaryotes. While *znxf-1* and *wago-4* mutants responded normally to exogenous RNAi against germline-expressed genes, their progeny were unable to inherit the silencing response. Moreover, ZNFX-1 colocalizes with WAGO-4 and CSR-1 in adult germ cells, but they form distinct foci from P granules and Mutator foci [170,180]. To reflect the unique identity of the ZNFX-1 and WAGO-4 foci, one group coined them Z granules and proposed the existence of PZM (P granule/Z granule/Mutator foci) tri-condensate assemblages in the adult germ cells [170]. Because components of the Mutator foci and P granules were previously found to affect RNAi inheritance [53,73,94], PZM assembly was deemed as required for the epigenetic inheritance of exogenous RNAi silencing [170]. 

A subsequent study conducted by Ouyang and colleagues asked whether Z granules function with the germline-specific and nuclear-localized HRDE-1, the chief effector in the inheritance of RNAi-induced transcriptional silencing states [71,188]. Using fluorescent in situ hybridization (FISH) to visualize the germline-specific *mex-6* RNA in hermaphrodites, the authors fed adult worms a *mex-6* dsRNA trigger and spatiotemporally tracked what happened to *mex-6* [188]. A reduction in the cytoplasmic *mex-6* signal was detected 4 h after initiating RNAi, feeding followed by an increase in the *mex-6* signal 2–4 h later in the nuclear puncta (i.e., nascent transcripts) of the pachytene and diplotene regions in the gonad, where meiotic prophase occurs. They also observed an aggregation of *mex-6* RNA clusters in the cytoplasm of developing oocytes that overlapped with PRG-1 (P granules), MUT-16 (Mutator foci), and ZNFX-1. Although cytoplasmic- and nuclear-localized *mex-6* signals were reduced in the F_1_ and F_2_ descendants of P_0_ hermaphrodites fed *mex-6* RNAi, the perinuclear puncta that overlapped with PRG-1 and ZNFX-1 remained detectable in pachytene, suggesting that the nuclear export of the *mex-6* mRNA persisted in the germ granules of the F_1_ and F_2_ generations. HRDE-1 was found to be necessary for silencing *mex-6* in the nucleus of the P_0_ and F_1_ generations, but ZNFX-1 was required for the accumulation of *mex-6* germ granule signals. This suggested that the HRDE-1 nuclear RNAi machinery functioned independently of the cytoplasmic response by ZNFX-1 of corralling transcripts targeted for silencing in the germ granules of P_0_ and F_1_ animals. Moreover, ZNFX-1 was found to be required for the amplification of *mex-6* sncRNAs and *mex-6* transcripts with a pUG (the addition of untemplated UGs to the 3’ end of an RNA, or “pUGylation”. See Section 5.1) in the F_1_ but not the P_0_ generation. Collectively, this study posits a novel concept of two parallel sncRNA amplification loops being dependent on the nuclear HRDE-1 targeting nascent transcripts and ZNFX-1 aggregating targeted RNA in perinuclear condensates [188].

## 4. Examples of Epigenetic Inheritance Induced by Biotic and Abiotic Stress

### 4.1. Heat Stress

Given that one of the first descriptions of epigenetic phenomena resulted from Waddington’s experiments with heat stress [2], it is not surprising that high temperature can trigger TEI responses across organisms. In this section, we highlight the advances made in *C. elegans*, which are highly sensitive to heat stress, regarding the role of small RNAs in the ancestral memory of high temperature. Cultivating a *C. elegans* population for one generation (~3 days) at the mild heat stress of 25 °C, followed by a return to its standard growth temperature of 20 °C, resulted in altered expression in 20 genes that endured for at least four generations. This list of genes was highly enriched for oocyte-expressed genes that are known targets of piRNAs and HRDE-1-bound siRNAs [189]. Once PRG-1/Piwi and its associated piRNAs induce silencing, the production of WAGO-associated siRNAs is required in subsequent generations to maintain TEI [73,93,94,95,96,190,191]. Interestingly, the piRNA-targeted transcripts whose levels change upon the shift to an elevated temperature are more than 10 times more likely to be transmitted transgenerationally when the animals were returned to the normal growth temperature; thus, PRG-1-dependent germline small RNAs are predictors of the generational transmission of ancestral temperature stress. In addition, the heritable effect of temperature was abrogated in strains carrying mutations in the *mut-2/rde-3* and *mut-16* Mutator genes, which are required for siRNA production [184,185]. Altogether, this study was one of the first showing an ecological stimulus effectively triggering a transgenerational heritable change in gene expression that depended on the action of small RNAs. 

A broader genome-wide study examining the effect of heat stress across generations in *C. elegans* included transcriptomic analysis of wild-type and *hrde-1* mutant animals grown under a fluctuating regime of low (15 °C) and high (23 °C) temperatures for 12 generations. The authors identified 288 genes that were upregulated due to heat stress in *hrde-1* adults compared to the wild-type as nuclear RNAi-repressed heat-inducible genes (NHGs) [192]. A subset of 41 NHGs exhibited a > 2-fold heat stress-dependent increase in expression in *hrde-1* mutants compared to the wild-type, which were named “high-stringent” NHGs. While most of the high-stringent NHGs were protein-coding, approximately 40% overlapped with LTR (long terminal repeat) retrotransposon elements, indicating that one of the key functions of HRDE-1 is to hamper certain LTR retrotransposons from activating upon heat stress [192]. ChIP-Seq analysis of these NHG regions indicated a correlation of increased expression in *hrde-1* adults with increased RNA polymerase II occupation and decreased histone H3K9me3 heterochromatic modifications, demonstrating a connection between HRDE-1 and chromatin modification pathways. Two subsequent studies have also found a role for histone H3K9me3 modifications in the TEI of heat stress response via transgene expression and lifespan extension, although the connection to endo-siRNA pathways in those instances has not been thoroughly explored [181,193]. These results indicate that gene silencing due to ancestral temperature stress is transgenerational and that the germline nuclear RNAi pathway plays a role in this repression via chromatin remodeling. Together, these studies indicate siRNA-mediated regulation of histone H3K9me3 modifications in propagation of heat stress memory and the corresponding changes in gene expression and physiology.

An outstanding and controversial issue in the TEI field is whether the inheritance of ancestral experiences is adaptive to a species. Evolutionary biologists have derived a new “unified evolution theory” to incorporate the effects of TEI into how natural selection impacts populations [194,195]. This new theory seems especially relevant when TEI impacts mating behaviors. *C. elegans* is androdioecious, meaning it can self-propagate or outcross. However, exposure to stressful conditions in *C. elegans* and other species increases the frequency of outcrossing, and hence the genetic variability essential for increasing survival in environmental fluctuations [196,197]. Upon the depletion of a finite number of self-made sperm, aging hermaphrodites secrete a volatile sex pheromone which attracts males via the SRD-1 receptor in their AWA sensory neurons [198,199]. However, after continuously maintaining animals for 10-15 generations at 25 °C, adult hermaphrodites precociously secreted a male-attracting pheromone that resulted in increased male attraction and mating. Interestingly, the premature attraction of males to hermaphrodites was preserved for up to three generations following the shift back to 20 °C and required HRDE-1/AGO, indicating that the phenotype was siRNA-based [200]. By screening strains carrying mutations for genes with functions in various small RNA pathways, four additional mutant strains (*alg-5*, *dcr-1*, *prg-1*, and the *meg-3/4* double mutant) were found to prematurely produce the sex pheromone, but only the *meg-3/4* double mutant exhibited TEI of the increased attractiveness to males [200]. All of the RNA pathway proteins whose lesions result in precocious male attraction localize to germline-specific structures called P granules, which are cytoplasmic condensates that house RNA and RNA-interacting proteins and are required for germline maintenance and TEI [201]. P granules are acutely disrupted in the *meg-3/4* mutant and are smaller in wild-type animals grown at high temperature [202,203]. Analysis of the mRNA and small RNA levels in precociously attractive hermaphrodites cultivated at a high temperature revealed the enrichment of sperm-related genes, which is consistent with the disruption in the structural integrity of P granules [204]. Production of the sex pheromone is dependent on the lack of a sperm and egg fusion signal [198]; thus, these observations suggest that the premature attractiveness of worms grown at 25 °C may be due to inappropriate gene expression in the germ cells and the inability to reproduce [200]. By performing multi-generational mating competition experiments, the authors showed that the proportion of the *meg-3/4*-descendent lineage increased over seven generations, suggesting that the production of the sex pheromone transgenerationally increases mating of the *meg-3/4*-descendent lineage, leading to its increased frequency within a population [200]. Altogether, high temperature affects the production and inheritance of siRNAs, which can result in a generational shift in population structure due to a change in pheromone production and the attractiveness of hermaphrodites.

### 4.2. Nutritional Stress

Epigenetic marks, including DNA methylation, histone modifications, and small RNAs, depend on metabolites for their biogenesis, and thus, are ultimately tied to metabolism [205]. However, how the parental diet can affect the metabolism of offspring is less understood. It is clear from human famine data that maternal under-nutrition during gestation contributes to metabolic disorder in offspring, in some cases transgenerationally [206,207]. The effects of the common Western high-fat and high-sugar diet (WD) have also been shown to have transgenerational effects [208,209,210]. Eggs contain material from the maternal somatic tissue, such as proteins, lipids, and mRNAs, the latter of which can jumpstart embryonic development after fertilization and the initiation of maternal-to-zygotic transition (MZT) [211,212,213]. Additionally, for most mammals, embryonic development occurs entirely in utero, making the developing fetus susceptible to the effects of maternal over- or under-nutrition [214,215,216]. However, the paternal contribution to the offspring’s metabolic phenotype has only become to be largely appreciated in the last decade. In this section, we will focus on recent studies examining the effects of paternal stress and diet on the offspring’s metabolic phenotype in mammals.

Developing germ cells undergo massive reprogramming during development, erasing most epigenetic information. Sperm were previously thought to contribute very little beyond haploid DNA content to the zygote, much less any “experience” of the parental condition. Sperm contain proteins, lipids, and RNA that can influence gene expression in a developing embryo [130,158,217,218]. The fundamental steps of spermatogenesis begin with germ cell differentiation into spermatocytes via mitotic division and the production of haploid spermatids from the tetraploid primary spermatocytes via meiotic division. This is followed by spermiogenesis when motile spermatozoa are produced from spermatids. In *C. elegans*, spermatogenesis in hermaphrodites is restricted to the last larval stage (L4) prior to the onset of oogenesis at adulthood and occurs in the spermatheca of the gonads, while in mammals, spermatogenesis occurs in the male testes [219,220]. Studies have found that miRNAs and tRFs transfer from the epididymis to the spermatozoa in mice, showing a direct way to transfer information between the somatic tissue and germline in males [148,221].

While both sperm and oocytes harbor miRNAs, most of the evidence for miRNAs in intergenerational inheritance comes from studies of paternally inherited sperm [121]. Male mice exposed to chronic stress before breeding sired offspring that exhibited higher incidences of neuropsychiatric disorder due to the dysregulation of the hypothalamic–pituitary–adrenal (HPA) stress axis. Analysis of the miRNA population in the sires with reduced HPA stress axis reactivity revealed higher levels of nine miRNAs (miR-29c, miR-30a, miR-30c, miR-32, miR-193-5p, miR-204, miR-375, miR532-3p, and miR-69) [120]. Microinjection of these nine miRNAs into zygotes, which were later implanted into surrogate females and reared under normal conditions, reprised the reduced HPA stress axis reactivity of the paternally stressed sires [119]. Another example of intergenerational inheritance via sperm is the mouse trauma model of unpredictable maternal separation combined with unpredictable maternal stress (MSUS), which results in changes in behavioral traits and glucose metabolism across generations. Assessment of the small RNA population from MSUS F_1_ sperm showed decreased levels of piRNAs and several miRNAs (miR-375-3p, miR-375-5p, miR-200b-3p, miR-672-5p, and miR-466-5p), the latter of which could potentially target over 70 genes involved in the regulation of DNA, epigenetics, RNA binding, RNA processing, stress response, and metabolism. A causal nexus between the MSUS sperm small RNA pool and its consequence across generations was proposed when RNA extracted from MSUS male sperm was microinjected into wild-type fertilized oocytes, resulting in the recapitulation of the MSUS acquired traits [117].

Two types of diets have been recently investigated for their role in regulating offspring metabolism: a high-fat diet (HFD) and low-protein diet (LPD). The consumption of a HFD results in a predisposition to obesity and metabolic disorders across generations [222,223,224,225]. One of the first studies showing that paternal diet influenced offspring metabolism examined the consequences of males fed a HFD mated with females fed a control diet. The HFD males themselves exhibited increased body weight and adiposity compared to control males, as well as insulin resistance and decreased glucose tolerance. Among the offspring of HFD males, only the females showed a change in body weight, exhibiting a trend toward lower day-1 body weights compared to the controls and also developing impaired glucose tolerance and insulin secretion as adults [226]. DNA methylation was investigated as a potential mechanism for the inheritance of intergenerational metabolic memory; however, the evidence of a correlation between methylation states and metabolic phenotypes was not conclusive.

Subsequent studies of the heritable effects of a paternal HFD implicated non-coding RNAs as a mechanism of metabolic memory. First, one group importantly demonstrated that other factors, such as seminal fluid, were not necessary for the inheritance of metabolic disorders by using in vitro fertilization of gametes followed by implantation into control females. As described above, female offspring were more susceptible to obesity phenotypes from a paternal HFD, and the effects of maternal and paternal HFDs can be additive with respect to weight gain in the offspring [227]. The sufficiency of information from sperm to inherit metabolic disease was further demonstrated by injecting mouse embryos with RNA extracted from sperm taken from HFD males. Analysis of the testis RNAs from HFD and control diet mice indicated that over a dozen miRNAs were differentially expressed. Microinjection of one of most abundantly dysregulated miRNAs, miR-19b, into one-cell embryos from the control diet parents was sufficient to induce metabolic disease [118]. Additionally, transcriptomic analysis of HFD male testes found differential expression of some miRNAs and piRNAs, including miRNA let-7c, indicating that non-coding RNAs transmitted via sperm could be the epigenetic factor regulating metabolic phenotypes [118]. 

Contemporaneously, two additional studies found that tRF levels were altered in the sperm of HFD males in addition to miRNAs and piRNAs [127,228]. Interestingly, the injection of purified tRFs from HFD sperm into embryos could recapitulate the metabolic disorder phenotypes [127]. However, synthetically derived tRFs had no effect on the offspring, suggesting that modifications made to the tRFs are essential to their functions [127]. Indeed, deletion of the tRNA methyltransferase, DNMT2, abolished the inheritance of metabolic phenotypes from high-fat-diet males [229]. In addition, although male offspring of HFD males typically do not display metabolic phenotypes on control diets, their inheritance of altered levels of tRFs can occur up to the F3 generation via the male lineage [230]. Together, these results suggest that paternal high-fat diets can sex-specifically program offspring to perpetuate metabolic phenotypes via altered miRNA and tRFs levels in sperm.

Unlike the offspring of HFD males, the offspring of LPD males did not exhibit obesity phenotypes, but instead had upregulated expression of lipid and cholesterol biosynthesis genes in their livers [231]. In this study, modest changes in miRNA expression, including upregulation of let-7, and DNA methylation were observed in the offspring livers, but they were not correlated with the levels observed in sperm of LPD males [231]. A follow-up study focused on the mechanism of the above observations by sequencing small RNAs (<40 nt) isolated from the cauda sperm of males on low-protein or control diets. Although this study focused primarily on the most abundant small RNA species, tRFs, it is of note that let-7 was downregulated in the sperm from LPD males. A small candidate screen of the effects of abundant tRFs using embryonic stem cells found that ~70 genes were upregulated when a specific tRF, tRF-Gly-Gcc, was inhibited. These genes are known to be regulated by the MERVL retroelement and are expressed in preimplantation embryos. Strikingly, the increased abundance of tRF-Gly-Gcc in the LPD sperm correlated with the decreased expression of MERVL-regulated genes in the embryonic offspring of LPD males [157]. How regulation of these genes corresponds to metabolic phenotypes in adulthood is yet to be determined. Overall, these studies demonstrate that male diet can impact offspring metabolism, and that miRNAs and abundant tRF species inherited via sperm may have tissue-specific effects on gene expression in the next generation.

### 4.3. Pathogens

Before the discovery of RNA interference, many genes with RNAi functions were initially identified and characterized for their roles in promoting genome integrity via the suppression of transposon activity. Currently, we understand that RNAi pathways function to distinguish “self” versus “non-self” transcripts to protect the cells against foreign nucleic acids [65,232,233,234]. Naturally, these functions could be extended to external pathogens, such as viruses and bacteria. However, in *C. elegans*, the siRNA and behavioral responses toward pathogens, whether intergenerational or transgenerational, is dependent upon the nature and duration of parental exposure [190,235,236,237]. For example, presenting *Pseudomonas vranovensis* only during the parental generation restrains the survival benefit to only the next generation, while persistent exposure to the *P. vranovensis* pathogen for several generations can enhance the survival of descendants transgenerationally [236]. In addition, *C. elegans* mothers that experienced *Pseudomonas aeruginosa* PA14 in a 4-h training window gave rise to progeny with an increased attraction to PA14, while an 8-h training period resulted in progeny with a PA14 aversive reaction [237]. These observations are suggestive of the complex networks governing the inheritance process [238,239,240].

A series of recent studies have uncovered a mechanism by which the behavior of *P. aeruginosa* avoidance in *C. elegans* is transmitted transgenerationally, resulting from an inter-kingdom gene regulation event. Exposure to the pathogenic bacterium *P. aeruginosa* PA14 for 24 h during the transition to adulthood in the parental generation resulted in avoidance that persisted for four generations [190]. In *C. elegans*, the DAF-7 ligand of the transforming growth factor beta (TGF-β) pathway is typically expressed in the pair of ASI chemosensory neurons [241]. Upon exposure to PA14, the expression of *daf-7/*TGF-β in the ASI neurons increases and its expression in the ASJ sensory neuron pair is also activated [241]. The loss of increased *daf-7* expression in the ASI neurons does not impede the hermaphrodite’s evasion of PA14 but rather abrogates PA14 avoidance in the F_1_ progeny, while loss of *daf-7* expression in the ASJ pair had no effect. Moreover, the increased *daf-7* expression in the ASI neurons extended until the F_4_ generation, mirroring their transgenerational learned avoidance behavior [190]. Additionally, animals carrying a mutant allele of the nuclear Argonaute HRDE-1 exhibited normal attraction to PA14 but were defective in PA14 avoidance learning [190]. Small RNA sequencing from parental worms fed OP50 or PA14 revealed the differential expression of miRNAs (mostly upregulated) and piRNAs (mostly downregulated) upon exposure to PA14. While some of the differentially expressed miRNAs have previously been reported to play a role in PA14 avoidance [242,243,244,245,246], Piwi had yet to be identified as a mediator of the transgenerational inheritance of pathogen response. The F_1_ generation of *prg-1* mutants exhibited defective avoidance to PA14, likely due to the lack of increased *daf-7* expression in the ASI neurons, indicating that PRG-1 is required for the generational transmission of learned aversion behavior. 

Interestingly, small RNAs extracted from PA14 were sufficient to confer learned pathogen evasion for four generations. Using differential expression analysis to compare the sRNA of virulent PA14 and less-virulent PA14, the authors identified a single RNA of 137 nucleotides, referred to as P11, as necessary and sufficient for PA14 learned avoidance and transgenerational memory [191]. P11 had the most complementarity to *C. elegans* mRNA expressed from a gene homologous to the mammalian macoilin gene, *maco-1*, which encodes a member of a family of highly conserved and broadly expressed transmembrane proteins that are primarily expressed in the nervous system [247]. MACO-1 is expressed in the ASI and other neurons and functions in neuronal excitability, locomotion, thermotaxis, chemotaxis, and dauer formation [248,249,250]. How is the P11 signal transmitted across tissues and generations? Crude whole-worm lysates or the liquid culture media used to grow worms (conditioned media or CM) from the grand-progeny of PA14-trained worms could pass the learned memory of PA14 aversion to naïve animals [251]. Using density-fractionated F_2_ lysates coupled with electron microscopy, viral-like particles (VLPs) were identified that coincided with the induction of PA14 avoidance in naïve animals that endured into their F_4_ generation. These VLPs were of similar size to the capsids formed by *Cer1*, an age- and temperature-dependent retrotransposon related to the Gypsy/Ty3 family of retroelements that is mostly expressed in *C. elegans* germ cells [252]. While hermaphrodites with mutant *Cer1* were still capable of PA14 avoidance, the avoidance behavior was not inherited by the F_1_ generation. Interestingly, *Cer1* expression was not detected in the neurons, and the neuronal expression of *Cer1* did not rescue PA14 avoidance, suggesting that *Cer1* acts upstream of *daf-7* since the loss of Cer1 results in increased *daf-7* expression in the ASI neurons. While vertical transfer of pathogen memory requires Cer1, this transposon is also required for the horizontal transmission of the transgenerational memory from exposed to naïve worms. By using generational-specific RNAi knockdowns of *Cer1* and previously identified contributors of transgenerational epigenetic memory [191], the authors found that Cer1 is not uniquely required by the germline but that it functions more as a germline-to-soma courier of PA14 avoidance memory in every generation [251]. Taken as a whole, these results illustrate that *C. elegans* has repurposed a potentially detrimental retrotransposon as a vehicle to communicate the environmental pathogen status to their naïve relatives and provide survival and evolutionary advantages [251].

## 5. Progress on Open Questions in Epigenetic Inheritance Biology

**Figure 2:**
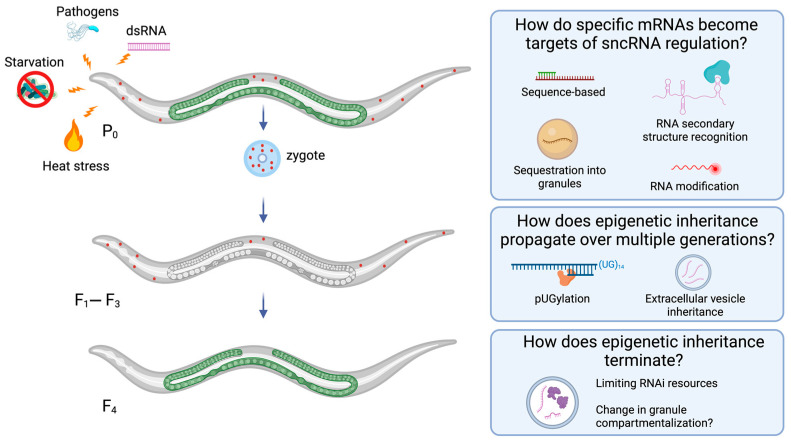
Open questions in sncRNA-mediated epigenetic inheritance. Environmental stresses experienced by *C. elegans* can trigger production of an RNAi response (red dots) that can silence gene expression in the germline (green) or the soma for multiple generations. Possible mechanisms regulating how mRNAs become targets, how RNAi is propagated over generations, and how epigenetic inheritance is terminated are illustrated in the blue boxes.

The idea of the long-lasting inheritance of acquired traits being heritable across multiple generations in animals is conceptually difficult to reconcile given the established knowledge that gametes undergo robust and extensive chromatin reprogramming. Epigenetic modifications regulating the parental genome, such as DNA methylation and histone modification, are erased so that their zygotic product is reset to its pluripotent state. However, a decade ago, several studies showed that environmental RNAi in *C. elegans* could establish an epigenetic memory inherited for multiple generations in the absence of the primary trigger. Using feeding RNAi against germline-expressed reporter transgenes with fluorescent tags, investigators demonstrated that a stable form of epigenetic inheritance was dependent upon initial silencing by piRNAs, followed by secondary siRNA production and nuclear RNAi via HRDE-1. Additionally, the putative histone H3K9 methyltransferases (HMTs), SET-32 and SET-25, were found to be required starting in the F1 progeny to maintain the silencing transgenerationally [73,93,94]. Collectively, these studies prompted the shift to more mechanistic questions of how particular mRNAs become targets of RNAi silencing, how gene expression states become re-established in the next generation, and how epigenetic inheritance terminates (Figure 2). In this section, we highlight findings that provide insights regarding these open questions in the understanding of the molecular mechanisms of transgenerational epigenetic inheritance.

### 5.1. How Do Specific mRNAs Become Targets of sncRNA Regulation?

During environmental or feeding RNAi, animals ingest bacteria that contain dsRNA with sequences homologous to the intended mRNA target in *C. elegans*. Once ingested, the dsRNA is imported into cells by the systemic RNAi machinery and spreads throughout the animal. All cells except in the neurons express the dsRNA import channel, SID-1, which preferentially imports dsRNA into the cells to feed into the exogenous RNAi pathway [54,253,254]. Mutations in the SID-1 gene result in a lack of gene silencing in the dsRNA-treated animal as well as its progeny [54,255]. DsRNA crosses the somatic barrier into the germline via SID-1, but also non-specifically through the vitellogenin or yolk lipoprotein receptor protein, RME-2 [256,257]. SID-1 homologs have been identified in numerous other species of invertebrates and vertebrates, including humans [55,258,259,260,261,262]. The observation that human SIDT1 facilitates the bidirectional transfer of dsRNA in the cell culture leads to the intriguing hypothesis that the systemic spread of RNAi signals from the soma to the germline may be a potential mechanism of epigenetic inheritance in mammals [259].

Once dsRNA triggers systemic RNAi in *C. elegans* animals, how is silencing of a target gene maintained in subsequent generations? Recent results suggest that targets of RNAi silencing become “marked” by untemplated nucleotides. First, PUP-1/CDE-1/CID-1 is a poly-U polymerase that adds untemplated uridines to the 3’ ends of targeted mRNAs and small RNAs [263]. PUP-1 is required for the inheritance of gene silencing resulting from environmental RNAi and stabilizes the interaction of siRNAs with WAGO-4, which is a cytoplasmic Argonaute required for TEI upstream of HRDE-1 [182,264]. For RNAe to occur, nuclear RNAi via HRDE-1 must promote the production of tertiary siRNAs, which allow maintenance of trigger-independent silencing for many generations [73,265]. The inheritance of these siRNAs appears to precede the formation of heterochromatin at the targeted locus in each generation [266].

Second, another untemplated dinucleotide, UG, has also been recently implicated in TEI [267]. In an unbiased screen to identify enzymes that add untemplated nucleotides to RNAs, a *C. elegans* RNAi protein, RDE-3/MUT-2, was found to add stretches of untemplated UG dinucleotides, or poly(UG), to the 3’ end of transposon and protein-coding mRNAs [267,268]. RDE-3/MUT-2 is homologous to other ribonucleotidyltransferases and was initially identified as necessary for Tc1 transposon silencing in *C. elegans* and later as part of the RNAi pathway [269,270,271,272]. The addition of at least eight UGs to the 3’ end of an RNA, or “pUGylation”, functions to mark mRNAs as targets of RdRPs [267,273]. The stretches of UGs form a G-quadraplex structure that is bound by the RdRP RRF-1, which produces anti-sense 22G secondary siRNAs of mRNA targets [273,274]. These pUGylated RNA fragments promote TEI in *C. elegans*, and de novo pUGylation of targets occurring in each generation is required for the maintenance of silencing [267]. What remains to be determined is how pUGylation may be used by endogenous RNAi pathways to respond to environmental stress, and whether this mechanism is conserved in other animals (Figure 2). Additionally, considerable evidence now suggests that G-quadraplex structures in DNA and RNA have functional roles in transcription, translation, telomere biology, and genome stability [275]. A recent study found that cellular stress in human cells promotes the formation of RNA G-quadruplexes [276], highlighting a need for investigation of how RNA secondary structures may play a role in mRNA targeting for post-translational regulation.

pUGylation likely plays a role in transgenerational silencing by promoting target mRNAs to localize to perinuclear granules where siRNAs are generated [267]. In *C. elegans*, multiple germ granules in seemingly distinct domains play a role in sncRNA gene regulation and inheritance (reviewed in Section 3). In germ cells, P granules localize to the nuclear pores [277]. They contain numerous RNAs and RNA-binding proteins with intrinsically disordered domains and numerous RNAi inheritance factors, including AGOs [278]. Mutator foci are adjacent to P granules and are where endo-siRNAs are generated [185]. RDE-3/MUT-2 and RRF-1 RdRP both localize to the Mutator focus, making it the likely location of de novo pUGylation and the amplification of the siRNA of pUGylated targets [279]. Other granules, such as the Z granule and SIMR focus, are predicted to facilitate RNAi inheritance and piRNA targeting, respectively [171,180]. While these granules are considered “hubs” of sncRNA regulation of gene expression, how they function together to sort and regulate mRNA targets, or whether their compartmentalization has evolved to limit their function, is an area of intense study (see Section 3) (Figure 2) [169,280].

### 5.2. How Does Epigenetic Inheritance Propagate over Generations?

Examples of epigenetic inheritance of parental responses to environmental stresses are abundant across the animal kingdom [19,25,26,28,29,237,281,282]. In humans, a number of pathological conditions stem from the inheritance of detrimental ancestral experiences driving phenotypes that are favorable under specific stressful conditions but are incongruous with the current environment [6,283,284,285,286,287,288]. In *C. elegans*, epigenetic inheritance is commonly triggered by exogenous dsRNA and typically lasts for one to four generations [289]. With continuous selection, however, the heritable response to RNAi can endure for more than 80 generations [290]. This exceptionally long perdurance of RNAi cannot simply be explained by the inheritance of RNAi factors by each generation since *C. elegans* produces ~250–300 progeny per generation, resulting in a dilution of ~4–8 billion in just four generations. What factors determine whether an epigenetic event is forgotten or preserved, and is it possible to mitigate those factors to prevent human diseases?

Studies in *C. elegans* support a model where the role of histone modifications in transgenerational inheritance is dependent on the context. As described above, the putative histone H3K9 methyltransferases (HMTs), SET-32 and SET-25, were found to act downstream, and non-redundantly, of piRNAs to maintain silencing transgenerationally [73,93,94]. However, subsequent studies determined that SET-25 and SET-32 were required for the onset, but not maintenance, of transgenerational silencing triggered by environmental RNAi against germline-specific genes, and for the establishment of silencing of certain HRDE-1 endogenous targets [291,292]. In another study, SET-32 and SET-25 were required for the onset of silencing triggered by anti-*gfp* dsRNA in the parental generation expressing a germline-expressed *pie-1::gfp::h2b* transgene but not for the maintenance of silencing in subsequent generations. The maintenance of *gfp* silencing was mediated by HRDE-1 [293]. Interestingly, mutations in *set-32* or the H3K9me2 HMT gene, *met-2*, result in a mortal germline phenotype (Mrt), whereby fertility is progressively lost over generations [264,293,294,295]. The Mrt phenotype is likely due to the unchecked biogenesis of the initial parental small RNA population and the aberrant accumulation of heritable small RNAs over generations [296]. Consistent with this hypothesis, the loss of HRDE-1 in the *met-2* mutant fully rescued both the Mrt phenotype and the stable heritable RNAi responses [296]. MET-2 and the SPR-5 histone H3K4me2 demethylase were previously found to reset the epigenetic ground state of the germline that is necessary to sustain germline immortality across generations [297]; thus, the generational endurance of RNAi silencing appears to be a calibrated process involving both germline chromatin and inherited small RNA populations. Additionally, histone H3K9 methylation may serve to indirectly restrain the transgenerational inheritance of gene silencing perpetuated by HRDE-1.

How does unchecked biogenesis of endo-siRNAs result in a mortal germline phenotype? Within the population of inherited sncRNAs from the hermaphrodite are endo-siRNAs that align anti-sense with a subset of endo-siRNA biogenesis genes. This observation suggests that RNAi mechanisms regulate the expression of genes required for its own function, creating a feedback loop to regulate RNAi perdurance [238,298]. Exogenously triggering RNAi with dsRNA homologous to different gene targets during different generations results in heightened RNAi responses to the ancestral triggers [238]. An important aspect of this work demonstrated that mutations that inhibit the endogenous RNAi pathway could alter the number of generations for which the exogenous RNAi trigger persisted, providing evidence for a previous hypothesis that endogenous and exogenous siRNA pathways compete for a limited number of cellular resources, such as RNAi proteins common to both pathways (Figure 2) [238,299]. Together, these results support a model where the active regulation of RNAi responses determines their inheritance or erasure.

Variability in the strength of the RNAi feedback loop likely contributes to the differences in the number of generations that the gene silencing lasts amongst genetically identical worms [238,289,290]. By following the lineages of animals expressing an integrated single copy of a *gfp* transgene expressed in the germ line, the authors were able to determine three rules that predict the duration of RNAi silencing. First, RNAi silencing initiated in a single mother was passed on equally to all her descendants, but there was considerable variability in the extent of RNAi silencing between mothers [239]. Second, some individuals have more potent and heritable RNAi silencing, regardless of the RNAi trigger, which may be connected to the extent to which endo-siRNA biogenesis factors are regulated. The third rule is a natural extension of the second: the more generations for which an RNAi response lasts, the more likely that response is acquired by the next generation. To determine the molecular mechanism regulating the heritable variability in RNAi responses, RNA-Seq was performed on animals with strong versus weak RNAi inheritance. Notably, heat shock genes, including the master regulator of the heat shock response, HSF-1, exhibited correlated expression between the different RNAi states [300,301]. HSF-1 has also been reported to be involved in small RNA metabolism and phenotypic plasticity [302,303,304]. Taken together, this study demonstrates how a mother’s stochastic gene expression state is a critically important determinant for various small RNA-dependent functions, including the transgenerational inheritance of RNAi silencing states due to exogenous RNAi or natural stresses [239].

### 5.3. How Does Epigenetic Inheritance Terminate?

One of the characteristics of epigenetic gene regulation is that the altered gene expression state eventually reverts to its original expression level. Presumably, inherited RNAi responses function to provide an adaptive response to an environmental stimulus via changes in gene expression and the corresponding phenotype. Given that RNAi responses are likely costly to maintain, it seems important for animals to evolve an active, not passive, mechanism to control how long inheritance lasts. Once established, how does TEI terminate to “reset” to its original gene expression program? 

If a sustained RNAi response is to provide adaptation to the organism in a particular stressful environment, it follows that changing the environment during an RNAi response could affect its ability to propagate the original response across generations. To test this hypothesis, *C. elegans* strains carrying germline-expressed *gfp* reporters were fed exogenous *gfp* dsRNA in the parental generation, followed by one of three stressors in the F_1_ generation: heat shock for 2 h, growth under hyperosmotic conditions for 2 days, or growth in the absence of food for 6 days. A strong heritable silencing response was found in the stressed F_1_ generation, but it was gradually “reset” in the unstressed generations up to F_5_. [240]. Interestingly, however, endogenously expressed sncRNAs are “reset” by stress for only one generation before re-establishing gene silencing states. Using a reverse genetics approach, the authors were able to identify the p38 mitogen-activated protein kinase (MAPK) pathway and SKN-1/Nrf2 transcription factor as necessary for stress-induced resetting of RNAi inheritance [240]. Stress responses to DNA damage, axonal injury, starvation, heat shock, osmotic stress, and innate immunity converge on the evolutionarily conserved MAPK signaling cascade [305], which regulates the nuclear localization of SKN-1to target genes with functions in stress response, homeostasis, and lifespan [306]. Perhaps not surprisingly, a subset of RNAi genes, including genes encoding the AGO NRDE-3 and RdRP RRF-3, were downregulated after stress in a SKN-1-dependent manner, connecting the activation of the stress response to the regulation of RNAi responses in *C. elegans* [240].

Several lines of evidence have recently suggested that the termination of epigenetic inheritance is a genetically regulated process. First, piRNAs are capable of triggering TEI for more than 20 generations [73], but new results suggest that PRG-1 may also erase TEI memory. As described above, piRNAs are only required for the initiation and not the continued silencing of gene expression, so how might PRG-1 contribute to the elimination of TEI perpetuated by 22G siRNAs? The first evidence that PRG-1 is required to “reset” the RNAi-based memory of gene expression was shown by our laboratory. Adult *C. elegans* animals that transiently passed through the dauer stage due to early-life starvation exhibited low levels of intestinally stored lipids compared to continuously developed adults. The F_1_ progeny of the postdauer adults, however, exhibited an increased amount of intestinal lipids compared to the F_1_ progeny of controls. In the F_2_ generation, the grand-progeny of the postdauer and control adults no longer showed any significant difference, indicating that the ancestral starvation memory was inherited for a single generation before resetting back to the original state [307]. By performing the same assay in RNAi mutants, we found that mutations in HRDE-1 eliminated the inheritance of the starvation memory, as measured by the intestinal lipid storage levels. The same assay revealed that mutations in PRG-1 did not affect the inheritance of the starvation memory, as expected, but instead perpetuated the memory to the F_2_ generation [307]. The number of generations the memory lasts beyond the F_2_ generation is unknown, but this study provided evidence that the duration of endogenous RNAi-dependent phenotypes could be modulated by PRG-1.

In parallel, another group performed a forward genetic screen to identify factors that limit TEI in *C. elegans*. The screen utilized a strain carrying a germline-expressed *gfp*::*h2b* reporter and a temperature-sensitive, gain-of-function allele of the *oma-1* gene that results in embryonic arrest. The strain was fed dsRNA homologous to both *gfp* and *oma-1*, which resulted in viable worms that did not express GFP in the germline for 4 to 10 generations once the RNAi trigger was removed. While most mutant strains identified in this screen extended the *gfp* silencing for an additional seven generations, mutant alleles in *prg-1* resulted in indefinite TEI, or “perpetual silencing”, after hundreds of generations [279]. The authors found that mutant *prg-1* populations established one of two epigenetic states after RNAi: 100% of animals either restored their native gene expression or remained in a state of perpetual silencing. Crosses to introduce or remove wild-type *prg-1* from a strain supported the model that PRG-1 acts to inhibit TEI maintenance and does not act early to establish perpetual silencing. In addition, these crosses demonstrated the ability of silenced genes (both *gfp* and *oma-1*) to paramutate, or silence in *trans*, expressed alleles of the same genes, suggesting that inheritance of small RNAs across generations promoted the perpetual silencing. Indeed, siRNAs targeting *oma-1* and *gfp* were detected in the perpetually silenced strains, and factors known to promote RNAi inheritance, such as HRDE-1, ZNFX-1, and RDE-3/MUT-2 (see above), were also required for persistent TEI [279]. In addition, a previous screen for *heri* (*h*eritable *e*nhancer of *R*NA*i*) mutants identified HERI-1, a protein with a chromodomain and a putative serine/threonine pseudokinase domain, as a negative regulator of persistent RNAi potentially acting downstream of HRDE-1, connecting the RNAi and histone modification pathways together in the regulation of epigenetic inheritance [298].

How do the piRNA, pUGylation, nuclear RNAi, and histone modification pathways interact to coordinate the termination of epigenetic inheritance? The overwhelming evidence suggests that the inheritance of gene regulation states is coordinated in the germline P granules [280]. The absence of germline P granules results in the ectopic expression of somatic genes in the germ line [308,309], and a recent study showed that P granules are important for the ability of RNAi components, particularly PRG-1 and HRDE-1, to distinguish “self” from “non-self” transcripts exiting the nucleus [310]. Thus, it seems likely that P granules function in the germ line to facilitate which transcripts should be expressed, which should be silenced, and which should be transgenerationally silenced. Proteins required for P granule assembly, such as DEPS-1, are also required for transgenerational inheritance to be triggered by exogenous RNAi [170,238,311]. DEPS-1 and RDE-3/MUT-2, required for pUGylation, have many overlapping gene targets [311]. Furthermore, DEPS-1 physically interacts with PRG-1 in the P granule and is required for piRNA-dependent silencing [312]. The piRNA pathway is thought to facilitate the recognition of “self” versus “non-self” transcripts [65,232], and failure of P granule formation results in a disruption of this process [310]. Tethering of an mRNA transcript to the P granule component PGL-1 results in its silencing [179]; thus, an open question in the field is whether the P granule proteins recognize and target specific transcripts that are “non-self” for downstream processes or whether they function to concentrate the RNAi machinery necessary to make that decision via a sequence-based mechanism. RNA modifications and post-translational modifications to AGOs are known to play a role in AGO affinity to particular siRNAs, but how particular RNAs are targeted for modification is still unknown [313]. While the model for RNAi inheritance appears to parallel the classic “chicken and the egg” paradox, future work to characterize mechanisms for how PRG-1 shifts its preference for silencing will likely yield insights into how establishment versus termination of gene silencing is achieved (Figure 2).

## 6. Conclusions

The ease with which RNAi is performed using the *C. elegans* model organism has propelled our understanding of gene regulation mechanisms by sncRNAs. Numerous experiments have exploited RNAi-by-feeding experiments targeting *gfp* or other transgenic reporters in forward genetic screens to identify the proteins playing a role in RNAi pathways. While this approach has been fruitful, these screens may not fully represent how endogenous RNAi occurs in response to environmental stress. Exogenously provided dsRNA utilizes limiting cellular resources required for RNAi, disrupting the function of other endogenous RNAi pathways [299]. In addition, these experiments likely bypass the endogenous mechanism of mRNA targeting. For example, exogenously provided dsRNA will lead to the RNAi targeting of transcripts with homologous sequences via a sequence-specific-based mechanism (Figure 2), but how are endogenous mRNAs targeted during stress conditions when they are typically not targeted? While significant progress has been made in animals regarding sncRNA regulation of gene expression, future work using advanced molecular tools and high-resolution imaging will be needed to track the RNAi components and their endogenous mRNA targets at the subcellular level.

## Data Availability

No new data were created or analyzed in this study. Data sharing is not applicable to this article.

## Abbreviations

3′UTR	3′ untranslated region
AGO	Argonaute
CM	conditioned media
dsRNA	double stranded RNA
endo-siRNA	endogenous siRNA
EV	extracellular vesicle
exo-siRNA	exogenous siRNA
FISH	fluorescent in situ hybridization
HFD	high fat diet
HMT	H3K9 methyltransferase
HPA	hypothalamic–pituitary–adrenal
LTR	long terminal repeat
LPD	low protein diet
MAPK	mitogen-activated protein kinase
miRNA	microRNA
Mrt	mortal germline phenotype
MSUS	maternal separation combined with unpredictable maternal stress
MZT	maternal-to-zygotic transition
NHG	nuclear RNAi-repressed heat-inducible gene
piRNA	Piwi interacting RNA
Piwi	p-element induced wimpy testis
RdRP	RNA dependent RNA polymerase
RNAe	RNA-induced epigenetic silencing
RNAi	RNA interference
SF1	superfamily one
siRNA	small interfering RNA
sncRNA	small non-coding RNA
snoRNA	small nucleolar RNA
TE	transposable element
TEI	transgenerational epigenetic inheritance
TGF-β	transforming growth factor beta
tiRNA	stress-induced transfer RNA-derived small RNA
tRF	transfer RNA-derived fragment
tRNA	transfer RNA
tsRNA	tRNA-derived small RNA
VLP	viral-like particles
WAGO	worm specific Argonaute

## References

[1] Waddington C.H. (1942). Canalization of Development and the Inheritance of Acquired Characters. Nature.

[2] Waddington C.H. (2012). The Epigenotype. Int. J. Epidemiol..

[3] MacDonald J.L., Tharin S., Hall S.E. (2022). Epigenetic Regulation of Nervous System Development and Function. Neurochem. Int..

[4] Bird A. (2007). Perceptions of Epigenetics. Nature.

[5] Cavalli G., Heard E. (2019). Advances in Epigenetics Link Genetics to the Environment and Disease. Nature.

[6] Heard E., Martienssen R.A. (2014). Transgenerational Epigenetic Inheritance: Myths and Mechanisms. Cell.

[7] Hubbard E.J.A., Schedl T. (2019). Biology of the *Caenorhabditis elegans* Germline Stem Cell System. Genetics.

[8] Skinner M.K. (2008). What Is an Epigenetic Transgenerational Phenotype?: F3 or F2. Reprod. Toxicol..

[9] Skinner M.K., Manikkam M., Guerrero-Bosagna C. (2010). Epigenetic Transgenerational Actions of Environmental Factors in Disease Etiology. Trends Endocrinol. Metab..

[10] Sun D., Maney D.L., Layman T.S., Chatterjee P., Yi S.V. (2019). Regional Epigenetic Differentiation of the Z Chromosome between Sexes in a Female Heterogametic System. Genome Res..

[11] Perez M.F., Lehner B. (2019). Vitellogenins—Yolk Gene Function and Regulation in *Caenorhabditis elegans*. Front. Physiol..

[12] Smil V. (1999). China’s Great Famine: 40 Years Later. BMJ.

[13] Ahmed F. (2010). Epigenetics: Tales of Adversity. Nature.

[14] Lumey L.H., Stein A.D., Susser E. (2011). Prenatal Famine and Adult Health. Annu. Rev. Public. Health.

[15] Tobi E.W., Slieker R.C., Luijk R., Dekkers K.F., Stein A.D., Xu K.M., Slagboom P.E., van Zwet E.W., Lumey L.H., Biobank-based Integrative Omics Studies Consortium (2018). DNA Methylation as a Mediator of the Association between Prenatal Adversity and Risk Factors for Metabolic Disease in Adulthood. Sci. Adv..

[16] Tobi E.W., van den Heuvel J., Zwaan B.J., Lumey L.H., Heijmans B.T., Uller T. (2018). Selective Survival of Embryos Can Explain DNA Methylation Signatures of Adverse Prenatal Environments. Cell Rep..

[17] Painter R.C., Osmond C., Gluckman P., Hanson M., Phillips D.I.W., Roseboom T.J. (2008). Transgenerational Effects of Prenatal Exposure to the Dutch Famine on Neonatal Adiposity and Health in Later Life. BJOG.

[18] Cheng Q., Trangucci R., Nelson K.N., Fu W., Collender P.A., Head J.R., Hoover C.M., Skaff N.K., Li T., Li X. (2020). Prenatal and Early-Life Exposure to the Great Chinese Famine Increased the Risk of Tuberculosis in Adulthood across Two Generations *Proc*. Natl. Acad. Sci. USA.

[19] Bowers M.E., Yehuda R. (2016). Intergenerational Transmission of Stress in Humans. Neuropsychopharmacology.

[20] Nomura Y., Rompala G., Pritchett L., Aushev V., Chen J., Hurd Y.L. (2021). Natural Disaster Stress during Pregnancy Is Linked to Reprogramming of the Placenta Transcriptome in Relation to Anxiety and Stress Hormones in Young Offspring. Mol. Psychiatry.

[21] Godfrey K.M., Lillycrop K.A., Burdge G.C., Gluckman P.D., Hanson M.A. (2007). Epigenetic Mechanisms and the Mismatch Concept of the Developmental Origins of Health and Disease. Pediatr. Res..

[22] Dantzer B., Goncalves I.B., Spence-Jones H.C., Bennett N.C., Heistermann M., Ganswindt A., Dubuc C., Gaynor D., Manser M.B., Clutton-Brock T.H. (2017). The Influence of Stress Hormones and Aggression on Cooperative Behaviour in Subordinate Meerkats. Proc. R. Soc. B Biol. Sci..

[23] Öst A., Lempradl A., Casas E., Weigert M., Tiko T., Deniz M., Pantano L., Boenisch U., Itskov P.M., Stoeckius M. (2014). Paternal Diet Defines Offspring Chromatin State and Intergenerational Obesity. Cell.

[24] Garbutt J.S., Little T.J. (2014). Maternal Food Quantity Affects Offspring Feeding Rate in Daphnia Magna. Biol. Lett..

[25] Zipple M.N., Archie E.A., Tung J., Altmann J., Alberts S.C. (2019). Intergenerational Effects of Early Adversity on Survival in Wild Baboons. eLife.

[26] Fallet M., Luquet E., David P., Cosseau C. (2020). Epigenetic Inheritance and Intergenerational Effects in Mollusks. Gene.

[27] Lamb S.D., Chia J.H.Z., Johnson S.L. (2020). Paternal Exposure to a Common Herbicide Alters the Behavior and Serotonergic System of Zebrafish Offspring. PLoS ONE.

[28] Bringer A., Cachot J., Dubillot E., Prunier G., Huet V., Clérandeau C., Evin L., Thomas H. (2022). Intergenerational Effects of Environmentally-Aged Microplastics on the Crassostrea Gigas. Environ. Pollut..

[29] Paul S.C., Singh P., Dennis A.B., Müller C. (2022). Intergenerational Effects of Early-Life Starvation on Life History, Consumption, and Transcriptome of a Holometabolous Insect. Am. Nat..

[30] Bline A.P., Le Goff A., Allard P. (2020). What Is Lost in the Weismann Barrier?. J. Dev. Biol..

[31] Cantone I., Fisher A.G. (2013). Epigenetic Programming and Reprogramming during Development. Nat. Struct. Mol. Biol..

[32] Atlasi Y., Stunnenberg H.G. (2017). The Interplay of Epigenetic Marks during Stem Cell Differentiation and Development. Nat. Rev. Genet..

[33] Eckersley-Maslin M.A., Alda-Catalinas C., Reik W. (2018). Dynamics of the Epigenetic Landscape during the Maternal-to-Zygotic Transition. Nat. Rev. Mol. Cell Biol..

[34] Greer E.L., Shi Y. (2012). Histone Methylation: A Dynamic Mark in Health, Disease and Inheritance. Nat. Rev. Genet..

[35] O’Kane C.J., Hyland E.M. (2019). Yeast Epigenetics: The Inheritance of Histone Modification States. Biosci. Rep..

[36] Saxton D.S., Rine J. (2019). Epigenetic Memory Independent of Symmetric Histone Inheritance. eLife.

[37] Tuscher J.J., Day J.J. (2019). Multigenerational Epigenetic Inheritance: One Step Forward, Two Generations Back. Neurobiol. Dis..

[38] Fitz-James M.H., Cavalli G. (2022). Molecular Mechanisms of Transgenerational Epigenetic Inheritance. Nat. Rev. Genet..

[39] Shan C.-M., Fang Y., Jia S. (2023). Leaving Histone Unturned for Epigenetic Inheritance. FEBS J..

[40] Zion E., Chen X. (2023). Studying Histone Inheritance in Different Systems Using Imaging-Based Methods and Perspectives. Biochem. Soc. Trans..

[41] Kim M., Costello J. (2017). DNA Methylation: An Epigenetic Mark of Cellular Memory. Exp. Mol. Med..

[42] Illum L.R.H., Bak S.T., Lund S., Nielsen A.L. (2018). DNA Methylation in Epigenetic Inheritance of Metabolic Diseases through the Male Germ Line. J. Mol. Endocrinol..

[43] Greeson K.W., Crow K.M.S., Edenfield R.C., Easley C.A. (2023). Inheritance of Paternal Lifestyles and Exposures through Sperm DNA Methylation. Nat. Rev. Urol..

[44] Kim V.N., Han J., Siomi M.C. (2009). Biogenesis of Small RNAs in Animals. Nat. Rev. Mol. Cell Biol..

[45] Ahringer J. (2006). Reverse genetics. The C. elegans Research Community.

[46] Billi A.C. (2014). Endogenous RNAi pathways in *C. elegans*. The C. elegans Research Community.

[47] Napoli C., Lemieux C., Jorgensen R. (1990). Introduction of a Chimeric Chalcone Synthase Gene into Petunia Results in Reversible Co-Suppression of Homologous Genes in Trans. Plant Cell.

[48] Fire A., Albertson D., Harrison S.W., Moerman D.G. (1991). Production of Antisense RNA Leads to Effective and Specific Inhibition of Gene Expression in *C. elegans* Muscle. Development.

[49] Izant J.G., Weintraub H. (1984). Inhibition of Thymidine Kinase Gene Expression by Anti-Sense RNA: A Molecular Approach to Genetic Analysis. Cell.

[50] Romano N., Macino G. (1992). Quelling: Transient Inactivation of Gene Expression in *Neurospora crassa* by Transformation with Homologous Sequences. Mol. Microbiol..

[51] Guo S., Kemphues K.J. (1995). Par-1, a Gene Required for Establishing Polarity in *C. elegans* Embryos, Encodes a Putative Ser/Thr Kinase That Is Asymmetrically Distributed. Cell.

[52] Fire A., Xu S., Montgomery M.K., Kostas S.A., Driver S.E., Mello C.C. (1998). Potent and Specific Genetic Interference by Double-Stranded RNA in *Caenorhabditis elegans*. Nature.

[53] Grishok A., Tabara H., Mello C.C. (2000). Genetic Requirements for Inheritance of RNAi in *C. elegans*. Science.

[54] Winston W.M., Molodowitch C., Hunter C.P. (2002). Hunter Systemic RNAi in *C. elegans* Requires the Putative Transmembrane Protein SID-1. Science.

[55] Duxbury M.S., Ashley S.W., Whang E.E. (2005). RNA Interference: A Mammalian SID-1 Homologue Enhances SiRNA Uptake and Gene Silencing Efficacy in Human Cells. Biochem. Biophys. Res. Commun..

[56] Miska E.A., Ferguson-Smith A.C. (2016). Transgenerational Inheritance: Models and Mechanisms of Non-DNA Sequence-Based Inheritance. Science.

[57] Rechavi O., Lev I. (2017). Principles of Transgenerational Small RNA Inheritance in *Caenorhabditis elegans*. Curr. Biol..

[58] Seroussi U., Li C., Sundby A.E., Lee T.L., Claycomb J.M., Saltzman A.L. (2022). Mechanisms of Epigenetic Regulation by *C. elegans* Nuclear RNA Interference Pathways. Semin. Cell Dev. Biol..

[59] Hutvágner G., McLachlan J., Pasquinelli A.E., Bálint É., Tuschl T., Zamore P.D. (2001). A Cellular Function for the RNA-Interference Enzyme Dicer in the Maturation of the Let-7 Small Temporal RNA. Science.

[60] Ketting R.F., Fischer S.E.J., Bernstein E., Sijen T., Hannon G.J., Plasterk R.H.A. (2001). Dicer Functions in RNA Interference and in Synthesis of Small RNA Involved in Developmental Timing in *C. elegans*. Genes. Dev..

[61] Hutvagner G., Simard M.J. (2008). Argonaute Proteins: Key Players in RNA Silencing. Nat. Rev. Mol. Cell Biol..

[62] Castel S.E., Martienssen R.A. (2013). RNA Interference in the Nucleus: Roles for Small RNAs in Transcription, Epigenetics and Beyond. Nat. Rev. Genet..

[63] Seroussi U., Lugowski A., Wadi L., Lao R.X., Willis A.R., Zhao W., Sundby A.E., Charlesworth A.G., Reinke A.W., Claycomb J.M. (2023). A Comprehensive Survey of *C. elegans* Argonaute Proteins Reveals Organism-Wide Gene Regulatory Networks and Functions. eLife.

[64] Youngman E.M., Claycomb J.M. (2014). From Early Lessons to New Frontiers: The Worm as a Treasure Trove of Small RNA Biology. Front. Genet..

[65] Almeida M.V., de Jesus Domingues A.M., Ketting R.F. (2019). Maternal and Zygotic Gene Regulatory Effects of Endogenous RNAi Pathways. PLoS Genet..

[66] Ghildiyal M., Zamore P.D. (2009). Small Silencing RNAs: An Expanding Universe. Nat. Rev. Genet..

[67] Claycomb J.M., Batista P.J., Pang K.M., Gu W., Vasale J.J., van Wolfswinkel J.C., Chaves D.A., Shirayama M., Mitani S., Ketting R.F. (2009). The Argonaute CSR-1 and Its 22G-RNA Cofactors Are Required for Holocentric Chromosome Segregation. Cell.

[68] Seth M., Shirayama M., Gu W., Ishidate T., Conte D., Mello C.C. (2013). The *C. elegans* CSR-1 Argonaute Pathway Counteracts Epigenetic Silencing to Promote Germline Gene Expression. Dev. Cell.

[69] Wedeles C.J., Wu M.Z., Claycomb J.M. (2013). Protection of Germline Gene Expression by the *C. elegans* Argonaute CSR-1. Dev. Cell.

[70] Cecere G., Hoersch S., O’Keeffe S., Sachidanandam R., Grishok A. (2014). Global Effects of the CSR-1 RNA Interference Pathway on the Transcriptional Landscape. Nat. Struct. Mol. Biol..

[71] Buckley B.A., Burkhart K.B., Gu S.G., Spracklin G., Kershner A., Fritz H., Kimble J., Fire A., Kennedy S. (2012). A Nuclear Argonaute Promotes Multigenerational Epigenetic Inheritance and Germline Immortality. Nature.

[72] Gu W., Shirayama M., Conte D., Vasale J., Batista P.J., Claycomb J.M., Moresco J.J., Youngman E.M., Keys J., Stoltz M.J. (2009). Distinct Argonaute-Mediated 22G-RNA Pathways Direct Genome Surveillance in the *C. elegans* Germline. Mol. Cell.

[73] Ashe A., Sapetschnig A., Weick E.-M., Mitchell J., Bagijn M.P., Cording A.C., Doebley A.-L., Goldstein L.D., Lehrbach N.J., Le Pen J. (2012). PiRNAs Can Trigger a Multigenerational Epigenetic Memory in the Germline of *C. elegans*. Cell.

[74] Vasale J.J., Gu W., Thivierge C., Batista P.J., Claycomb J.M., Youngman E.M., Duchaine T.F., Mello C.C., Conte D. (2010). Sequential Rounds of RNA-Dependent RNA Transcription Drive Endogenous Small-RNA Biogenesis in the ERGO-1/Argonaute Pathway. Proc. Natl. Acad. Sci. USA.

[75] Mills R.E., Bennett E.A., Iskow R.C., Devine S.E. (2007). Which Transposable Elements Are Active in the Human Genome?. Trends Genet..

[76] Chakraborty M., VanKuren N.W., Zhao R., Zhang X., Kalsow S., Emerson J.J. (2018). Hidden Genetic Variation Shapes the Structure of Functional Elements in Drosophila. Nat. Genet..

[77] Laricchia K.M., Zdraljevic S., Cook D.E., Andersen E.C. (2017). Natural Variation in the Distribution and Abundance of Transposable Elements across the *Caenorhabditis elegans* Species. Mol. Biol. Evol..

[78] Barrón M.G., Fiston-Lavier A.S., Petrov D.A., González J. (2014). Population Genomics of Transposable Elements in *Drosophila*. Annu. Rev. Genet..

[79] Jonathan N. (2020). Wells; Cedric Feschotte A Field Guide to Eukaryotic Transposable Elements. Annu. Rev. Genet..

[80] Feschotte C. (2008). Transposable Elements and the Evolution of Regulatory Networks. Nat. Rev. Genet..

[81] Senft A.D., Macfarlan T.S. (2021). Transposable Elements Shape the Evolution of Mammalian Development. Nat. Rev. Genet..

[82] Chuong E.B., Elde N.C., Feschotte C. (2017). Regulatory Activities of Transposable Elements: From Conflicts to Benefits. Nat. Rev. Genet..

[83] Kathleen H. (2020). Burns Our Conflict with Transposable Elements and Its Implications for Human Disease. Annu. Rev. Pathol. Mech. Dis..

[84] Wylie A., Jones A.E., D’Brot A., Lu W.-J., Kurtz P., Moran J.V., Rakheja D., Chen K.S., Hammer R.E., Comerford S.A. (2016). P53 Genes Function to Restrain Mobile Elements. Genes. Dev..

[85] Wylie A., Jones A.E., Abrams J.M. (2016). P53 in the Game of Transposons. BioEssays.

[86] Tiwari B., Jones A.E., Caillet C.J., Das S., Royer S.K., Abrams J.M. (2020). P53 Directly Represses Human LINE1 Transposons. Genes. Dev..

[87] Iwasaki Y.W., Siomi M.C., Siomi H. (2015). PIWI-Interacting RNA: Its Biogenesis and Functions. Annu. Rev. Biochem..

[88] Czech B., Munafò M., Ciabrelli F., Eastwood E.L., Fabry M.H., Kneuss E., Hannon G.J. (2018). PiRNA-Guided Genome Defense: From Biogenesis to Silencing. Annu. Rev. Genet..

[89] Ozata D.M., Gainetdinov I., Zoch A., O’Carroll D., Zamore P.D. (2019). PIWI-Interacting RNAs: Small RNAs with Big Functions. Nat. Rev. Genet..

[90] Picard G. (1976). Non-Mendelian Female Sterility in *Drosophila melanogaster*: Hereditary Transmission of I Factor. Genetics.

[91] Kidwell M.G., Kidwell J.F., Sved J.A. (1977). Hybrid Dysgenesis in *Drosophila melanogaster*: A Syndrome of Aberrant Traits Including Mutation, Sterility and Male Recombination. Genetics.

[92] Mérel V., Boulesteix M., Fablet M., Vieira C. (2020). Transposable Elements in Drosophila. Mob. DNA.

[93] Luteijn M.J., van Bergeijk P., Kaaij L.J.T., Almeida M.V., Roovers E.F., Berezikov E., Ketting R.F. (2012). Extremely Stable Piwi-Induced Gene Silencing in *Caenorhabditis elegans*. EMBO J..

[94] Shirayama M., Seth M., Lee H.-C., Gu W., Ishidate T., Conte D., Mello C.C. (2012). PiRNAs Initiate an Epigenetic Memory of Nonself RNA in the *C. elegans* Germline. Cell.

[95] Lee H.-C., Gu W., Shirayama M., Youngman E., Conte D., Mello C.C. (2012). *C. elegans* PiRNAs Mediate the Genome-Wide Surveillance of Germline Transcripts. Cell.

[96] Belicard T., Jareosettasin P., Sarkies P. (2018). The PiRNA Pathway Responds to Environmental Signals to Establish Intergenerational Adaptation to Stress. BMC Biol..

[97] Brennecke J., Aravin A.A., Stark A., Dus M., Kellis M., Sachidanandam R., Hannon G.J. (2007). Discrete Small RNA-Generating Loci as Master Regulators of Transposon Activity in Drosophila. Cell.

[98] Aravin A.A., Hannon G.J., Brennecke J. (2007). The Piwi-PiRNA Pathway Provides an Adaptive Defense in the Transposon Arms Race. Science.

[99] Klattenhoff C., Xi H., Li C., Lee S., Xu J., Khurana J.S., Zhang F., Schultz N., Koppetsch B.S., Nowosielska A. (2009). The Drosophila HP1 Homolog Rhino Is Required for Transposon Silencing and PiRNA Production by Dual-Strand Clusters. Cell.

[100] Mohn F., Sienski G., Handler D., Brennecke J. (2014). The Rhino-Deadlock-Cutoff Complex Licenses Noncanonical Transcription of Dual-Strand PiRNA Clusters in Drosophila. Cell.

[101] Andersen P.R., Tirian L., Vunjak M., Brennecke J. (2017). A Heterochromatin-Dependent Transcription Machinery Drives PiRNA Expression. Nature.

[102] Siomi M.C., Miyoshi T., Siomi H. (2010). PiRNA-Mediated Silencing in Drosophila Germlines. Semin. Cell Dev. Biol..

[103] Gebert D., Neubert L.K., Lloyd C., Gui J., Lehmann R., Teixeira F.K. (2021). Large Drosophila Germline PiRNA Clusters Are Evolutionarily Labile and Dispensable for Transposon Regulation. Mol. Cell.

[104] Lee R.C., Feinbaum R.L., Ambros V. (1993). The *C. elegans* Heterochronic Gene Lin-4 Encodes Small RNAs with Antisense Complementarity to Lin-14. Cell.

[105] Pasquinelli A.E., Reinhart B.J., Slack F., Martindale M.Q., Kuroda M.I., Maller B., Hayward D.C., Ball E.E., Degnan B., Müller P. (2000). Conservation of the Sequence and Temporal Expression of Let-7 Heterochronic Regulatory RNA. Nature.

[106] Ambros V., Ruvkun G. (2018). Recent Molecular Genetic Explorations of *Caenorhabditis elegans* MicroRNAs. Genetics.

[107] Bartel D.P. (2018). Metazoan MicroRNAs. Cell.

[108] Gebert L.F.R., MacRae I.J. (2019). Regulation of MicroRNA Function in Animals. Nat. Rev. Mol. Cell Biol..

[109] Chen T.-H., Chen J.-A. (2019). Multifaceted Roles of MicroRNAs: From Motor Neuron Generation in Embryos to Degeneration in Spinal Muscular Atrophy. eLife.

[110] Song X., Li Y., Cao X., Qi Y. (2019). MicroRNAs and Their Regulatory Roles in Plant-Enviornment Interactions. Annu. Rev. Plant Biol..

[111] Agbu P., Carthew R.W. (2021). MicroRNA-Mediated Regulation of Glucose and Lipid Metabolism. Nat. Rev. Mol. Cell Biol..

[112] Kozomara A., Birgaoanu M., Griffiths-Jones S. (2019). MiRBase: From MicroRNA Sequences to Function. Nucleic Acids Res..

[113] Ha M., Kim V.N. (2014). Regulation of MicroRNA Biogenesis. Nat. Rev. Mol. Cell Biol..

[114] Treiber T., Treiber N., Meister G. (2019). Regulation of MicroRNA Biogenesis and Its Crosstalk with Other Cellular Pathways. Nat. Rev. Mol. Cell Biol..

[115] Dexheimer P.J., Cochella L. (2020). MicroRNAs: From Mechanism to Organism. Front. Cell Dev. Biol..

[116] Das P.P., Bagijn M.P., Goldstein L.D., Woolford J.R., Lehrbach N.J., Sapetschnig A., Buhecha H.R., Gilchrist M.J., Howe K.L., Stark R. (2008). Piwi and PiRNAs Act Upstream of an Endogenous SiRNA Pathway to Suppress Tc3 Transposon Mobility in the *Caenorhabditis elegans* Germline. Mol. Cell.

[117] Gapp K., Jawaid A., Sarkies P., Bohacek J., Pelczar P., Prados J., Farinelli L., Miska E., Mansuy I.M. (2014). Implication of Sperm RNAs in Transgenerational Inheritance of the Effects of Early Trauma in Mice. Nat. Neurosci..

[118] Grandjean V., Fourré S., De Abreu D.A.F., Derieppe M.-A., Remy J.-J., Rassoulzadegan M. (2015). RNA-Mediated Paternal Heredity of Diet-Induced Obesity and Metabolic Disorders. Sci. Rep..

[119] Rodgers A.B., Morgan C.P., Leu N.A., Bale T.L. (2015). Transgenerational Epigenetic Programming via Sperm MicroRNA Recapitulates Effects of Paternal Stress. Proc. Natl. Acad. Sci. USA.

[120] Rodgers A.B., Morgan C.P., Bronson S.L., Revello S., Bale T.L. (2013). Paternal Stress Exposure Alters Sperm MicroRNA Content and Reprograms Offspring HPA Stress Axis Regulation. J. Neurosci..

[121] Sharma U. (2019). Paternal Contributions to Offspring Health: Role of Sperm Small RNAs in Intergenerational Transmission of Epigenetic Information. Front. Cell Dev. Biol..

[122] Wang Y., Chen Z.-P., Hu H., Lei J., Zhou Z., Yao B., Chen L., Liang G., Zhan S., Zhu X. (2021). Sperm MicroRNAs Confer Depression Susceptibility to Offspring. Sci. Adv..

[123] Corrêa R.L., Steiner F.A., Berezikov E., Ketting R.F. (2010). MicroRNA–Directed SiRNA Biogenesis in *Caenorhabditis elegans*. PLoS Genet..

[124] Speer J., Gehrke C.W., Kuo K.C., Waalkes T.P., Borek E. (1979). TRNA Breakdown Products as Markers for Cancer. Cancer.

[125] Levitz R., Chapman D., Amitsur M., Green R., Snyder L., Kaufmann G.J. (1990). The Optional *E. coli* Prr Locus Encodes a Latent Form of Phage T4-induced Anticodon Nuclease. EMBO J..

[126] Lee Y.S., Shibata Y., Malhotra A., Dutta A. (2009). A Novel Class of Small RNAs: TRNA-Derived RNA Fragments (TRFs). Genes. Dev..

[127] Chen Q., Yan M., Cao Z., Li X., Zhang Y., Shi J., Feng G., Peng H., Zhang X., Zhang Y. (2016). Sperm TsRNAs Contribute to Intergenerational Inheritance of an Acquired Metabolic Disorder. Science.

[128] Czech A., Wende S., Mörl M., Pan T., Ignatova Z. (2013). Reversible and Rapid Transfer-RNA Deactivation as a Mechanism of Translational Repression in Stress. PLoS Genet..

[129] Perez M.F., Lehner B. (2019). Intergenerational and Transgenerational Epigenetic Inheritance in Animals. Nat. Cell Biol..

[130] Park J., Ahn S.H., Shin M.G., Kim H.K., Chang S. (2020). TRNA-Derived Small RNAs: Novel Epigenetic Regulators. Cancers.

[131] Kumar P., Anaya J., Mudunuri S.B., Dutta A. (2014). Meta-Analysis of TRNA Derived RNA Fragments Reveals That They Are Evolutionarily Conserved and Associate with AGO Proteins to Recognize Specific RNA Targets. BMC Biol..

[132] Kumar P., Mudunuri S.B., Anaya J., Dutta A. (2015). TRFdb: A Database for Transfer RNA Fragments. Nucleic Acids Res..

[133] Weng Q., Wang Y., Xie Y., Yu X., Zhang S., Ge J., Li Z., Ye G., Guo J. (2022). Extracellular Vesicles-Associated TRNA-Derived Fragments (TRFs): Biogenesis, Biological Functions, and Their Role as Potential Biomarkers in Human Diseases. J. Mol. Med..

[134] Willis I.M., Moir R.D. (2018). Moir Signaling to and from the RNA Polymerase III Transcription and Procesing Machinery. Annu. Rev. Biochem..

[135] Dubrovsky E.B., Dubrovskaya V.A., Levinger L., Schiffer S., Marchfelder A. (2004). Drosophila RNase Z Processes Mitochondrial and Nuclear Pre-tRNA 3′ Ends in Vivo. Nucleic Acids Res..

[136] Jarrous N., Reiner R. (2007). Human RNase P: A TRNA-Processing Enzyme and Transcription Factor. Nucleic Acids Res..

[137] Maraia R.J., Lamichhane T.N. (2011). 3′ Processing of Eukaryotic Precursor TRNAs. WIREs RNA.

[138] Hopper A.K., Nostramo R.T. (2019). TRNA Processing and Subcellular Trafficking Proteins Multitask in Pathways for Other RNAs. Front. Genet..

[139] Su Z., Wilson B., Kumar P., Dutta A. (2020). Noncanonical Roles of TRNAs: TRNA Fragments and Beyond. Annu. Rev. Genet..

[140] Tuck A.C., Tollervey D. (2011). RNA in Pieces. Trends Genet..

[141] Magee R., Rigoutsos I. (2020). On the Expanding Roles of TRNA Fragments in Modulating Cell Behavior. Nucleic Acids Res..

[142] Zhang S., Yu X., Xie Y., Ye G., Guo J. (2023). TRNA Derived Fragments:A Novel Player in Gene Regulation and Applications in Cancer. Front. Oncol..

[143] Anderson P., Ivanov P. (2014). TRNA Fragments in Human Health and Disease. FEBS Lett..

[144] Keam S.P., Hutvagner G. (2015). TRNA-Derived Fragments (TRFs): Emerging New Roles for an Ancient RNA in the Regulation of Gene Expression. Life.

[145] Schorey J.S., Cheng Y., Singh P.P., Smith V.L. (2015). Exosomes and Other Extracellular Vesicles in Host–Pathogen Interactions. EMBO Rep..

[146] van Niel G., D’Angelo G., Raposo G. (2018). Shedding Light on the Cell Biology of Extracellular Vesicles. Nat. Rev. Mol. Cell Biol..

[147] Chiou N.-T., Kageyama R., Ansel K.M. (2018). Selective Export into Extracellular Vesicles and Function of TRNA Fragments during T Cell Activation. Cell Rep..

[148] Sharma U., Sun F., Conine C.C., Reichholf B., Kukreja S., Herzog V.A., Ameres S.L., Rando O.J. (2018). Small RNAs Are Trafficked from the Epididymis to Developing Mammalian Sperm. Dev. Cell.

[149] Kim H.K., Yeom J.-H., Kay M.A. (2020). Transfer RNA-Derived Small RNAs: Another Layer of Gene Regulation and Novel Targets for Disease Therapeutics. Mol. Ther..

[150] Wilson B., Dutta A. (2022). Function and Therapeutic Implications of TRNA Derived Small RNAs. Front. Mol. Biosci..

[151] Yu M., Lu B., Zhang J., Ding J., Liu P., Lu Y. (2020). TRNA-Derived RNA Fragments in Cancer: Current Status and Future Perspectives. J. Hematol. Oncol..

[152] Garcia-Silva M.R., Cabrera-Cabrera F., Cura das Neves R.F., Souto-Padrón T., de Souza W., Cayota A. (2014). Gene Expression Changes Induced by *Trypanosoma cruzi* Shed Microvesicles in Mammalian Host Cells: Relevance of TRNA-Derived Halves. BioMed Res. Int..

[153] Cooke W.R., Cribbs A., Zhang W., Kandzija N., Motta-Mejia C., Dombi E., Ri R., Cerdeira A.S., Redman C., Vatish M. (2019). Maternal Circulating Syncytiotrophoblast-Derived Extracellular Vesicles Contain Biologically Active 5’-TRNA Halves. Biochem. Biophys. Res. Commun..

[154] Gu C., Begley T.J., Dedon P.C. (2014). TRNA Modifications Regulate Translation during Cellular Stress. FEBS Lett..

[155] Kumar P., Kuscu C., Dutta A. (2016). Biogenesis and Function of Transfer RNA-Related Fragments (TRFs). Trends Biochem. Sci..

[156] Guzzi N., Bellodi C. (2021). Stressin’ and Slicin’: Stress-Induced TRNA Fragmentation Codon-Adapts Translation to Repress Cell Growth. EMBO J..

[157] Sharma U., Conine C.C., Shea J.M., Boskovic A., Derr A.G., Bing X.Y., Belleannee C., Kucukural A., Serra R.W., Sun F. (2016). Biogenesis and Function of TRNA Fragments during Sperm Maturation and Fertilization in Mammals. Science.

[158] Gapp K., Miska E.A. (2016). TRNA Fragments: Novel Players in Intergenerational Inheritance. Cell Res..

[159] Ender C., Krek A., Friedländer M.R., Beitzinger M., Weinmann L., Chen W., Pfeffer S., Rajewsky N., Meister G. (2008). A Human SnoRNA with MicroRNA-Like Functions. Mol. Cell.

[160] Brameier M., Herwig A., Reinhardt R., Walter L., Gruber J. (2011). Human Box C/D SnoRNAs with MiRNA like Functions: Expanding the Range of Regulatory RNAs. Nucleic Acids Res..

[161] He X., Chen X., Zhang X., Duan X., Pan T., Hu Q., Zhang Y., Zhong F., Liu J., Zhang H. (2015). An Lnc RNA (GAS5)/SnoRNA-Derived PiRNA Induces Activation of TRAIL Gene by Site-Specifically Recruiting MLL/COMPASS-like Complexes. Nucleic Acids Res..

[162] Zhong F., Zhou N., Wu K., Guo Y., Tan W., Zhang H., Zhang X., Geng G., Pan T., Luo H. (2015). A SnoRNA-Derived PiRNA Interacts with Human Interleukin-4 Pre-MRNA and Induces Its Decay in Nuclear Exosomes. Nucleic Acids Res..

[163] Deogharia M., Majumder M. (2019). Guide SnoRNAs: Drivers or Passengers in Human Disease?. Biology.

[164] Bratkovič T., Božič J., Rogelj B. (2020). Functional Diversity of Small Nucleolar RNAs. Nucleic Acids Res..

[165] Wajahat M., Bracken C.P., Orang A. (2021). Emerging Functions for SnoRNAs and SnoRNA-Derived Fragments. Int. J. Mol. Sci..

[166] Huang Z., Du Y., Wen J., Lu B., Zhao Y. (2022). SnoRNAs: Functions and Mechanisms in Biological Processes, and Roles in Tumor Pathophysiology. Cell Death Discov..

[167] Voronina E., Seydoux G., Sassone-Corsi P., Nagamori I. (2011). RNA Granules in Germ Cells. Cold Spring Harb. Perspect. Biol..

[168] Trcek T., Lehmann R. (2017). All about the RNA after All. eLife.

[169] Ouyang J.P.T., Seydoux G. (2022). Nuage Condensates: Accelerators or Circuit Breakers for SRNA Silencing Pathways?. RNA.

[170] Wan G., Fields B.D., Spracklin G., Shukla A., Phillips C.M., Kennedy S. (2018). Spatiotemporal Regulation of Liquid-like Condensates in Epigenetic Inheritance. Nature.

[171] Manage K.I., Rogers A.K., Wallis D.C., Uebel C.J., Anderson D.C., Nguyen D.A.H., Arca K., Brown K.C., Cordeiro Rodrigues R.J., de Albuquerque B.F. (2020). A Tudor Domain Protein, SIMR-1, Promotes SiRNA Production at PiRNA-Targeted MRNAs in *C. elegans*. eLife.

[172] Sundby A.E., Molnar R.I., Claycomb J.M. (2021). Connecting the Dots: Linking *Caenorhabditis elegans* Small RNA Pathways and Germ Granules. Trends Cell Biol..

[173] Batista P.J., Ruby J.G., Claycomb J.M., Chiang R., Fahlgren N., Kasschau K.D., Chaves D.A., Gu W., Vasale J.J., Duan S. (2008). PRG-1 and 21U-RNAs Interact to Form the PiRNA Complex Required for Fertility in *C. elegans*. Mol. Cell.

[174] Wang G., Reinke V. (2008). A *C. elegans* Piwi, PRG-1, Regulates 21U-RNAs during Spermatogenesis. Curr. Biol..

[175] Conine C.C., Batista P.J., Gu W., Claycomb J.M., Chaves D.A., Shirayama M., Mello C.C. (2010). Argonautes ALG-3 and ALG-4 Are Required for Spermatogenesis-Specific 26G-RNAs and Thermotolerant Sperm in *Caenorhabditis elegans*. Proc. Natl. Acad. Sci. USA.

[176] Updike D., Strome S. (2010). P Granule Assembly and Function in *Caenorhabditis elegans* Germ Cells. J. Androl..

[177] Updike D.L., Hachey S.J., Kreher J., Strome S. (2011). P Granules Extend the Nuclear Pore Complex Environment in the *C. elegans* Germ Line. J. Cell Biol..

[178] Brown K.C., Svendsen J.M., Tucci R.M., Montgomery B.E., Montgomery T.A. (2017). ALG-5 Is a MiRNA-Associated Argonaute Required for Proper Developmental Timing in the *Caenorhabditis elegans* Germline. Nucleic Acids Res..

[179] Aoki S.T., Lynch T.R., Crittenden S.L., Bingman C.A., Wickens M., Kimble J. (2021). *C. elegans* Germ Granules Require Both Assembly and Localized Regulators for MRNA Repression. Nat. Commun..

[180] Ishidate T., Ozturk A.R., Durning D.J., Sharma R., Shen E., Chen H., Seth M., Shirayama M., Mello C.C. (2018). ZNFX-1 Functions within Perinuclear Nuage to Balance Epigenetic Signals. Mol. Cell.

[181] Wan Q.-L., Meng X., Dai W., Luo Z., Wang C., Fu X., Yang J., Ye Q., Zhou Q. (2021). N6-Methyldeoxyadenine and Histone Methylation Mediate Transgenerational Survival Advantages Induced by Hormetic Heat Stress. Sci. Adv..

[182] Xu F., Guang S., Feng X. (2018). Distinct Nuclear and Cytoplasmic Machineries Cooperatively Promote the Inheritance of RNAi in *Caenorhabditis elegans*. Biol. Cell.

[183] Placentino M., de Jesus Domingues A.M., Schreier J., Dietz S., Hellmann S., de Albuquerque B.F., Butter F., Ketting R.F. (2021). Intrinsically Disordered Protein PID-2 Modulates Z Granules and Is Required for Heritable PiRNA-Induced Silencing in the *Caenorhabditis elegans* Embryo. EMBO J..

[184] Zhang C., Montgomery T.A., Gabel H.W., Fischer S.E.J., Phillips C.M., Fahlgren N., Sullivan C.M., Carrington J.C., Ruvkun G. (2011). Mut-16 and Other Mutator Class Genes Modulate 22G and 26G SiRNA Pathways in *Caenorhabditis elegans*. Proc. Natl. Acad. Sci. USA.

[185] Phillips C.M., Montgomery T.A., Breen P.C., Ruvkun G. (2012). MUT-16 Promotes Formation of Perinuclear Mutator Foci Required for RNA Silencing in the *C. elegans* Germline. Genes. Dev..

[186] Tsai H.-Y., Chen C.-C.G., Conte D., Moresco J.J., Chaves D.A., Mitani S., Yates J.R., Tsai M.-D., Mello C.C. (2015). A Ribonuclease Coordinates SiRNA Amplification and MRNA Cleavage during RNAi. Cell.

[187] Uebel C.J., Anderson D.C., Mandarino L.M., Manage K.I., Aynaszyan S., Phillips C.M. (2018). Distinct Regions of the Intrinsically Disordered Protein MUT-16 Mediate Assembly of a Small RNA Amplification Complex and Promote Phase Separation of Mutator Foci. PLoS Genet..

[188] Ouyang J.P.T., Zhang W.L., Seydoux G. (2022). The Conserved Helicase ZNFX-1 Memorializes Silenced RNAs in Perinuclear Condensates. Nat. Cell Biol..

[189] Schott D., Yanai I., Hunter C.P. (2014). Natural RNA Interference Directs a Heritable Response to the Environment. Sci. Rep..

[190] Moore R.S., Kaletsky R., Murphy C.T. (2019). Piwi/PRG-1 Argonaute and TGF-β Mediate Transgenerational Learned Pathogenic Avoidance. Cell.

[191] Kaletsky R., Moore R.S., Vrla G.D., Parsons L.R., Gitai Z., Murphy C.T. (2020). *C. elegans* Interprets Bacterial Non-Coding RNAs to Learn Pathogenic Avoidance. Nature.

[192] Ni J.Z., Kalinava N., Chen E., Huang A., Trinh T., Gu S.G. (2016). A Transgenerational Role of the Germline Nuclear RNAi Pathway in Repressing Heat Stress-Induced Transcriptional Activation in *C. elegans*. Epigenet. Chromatin.

[193] Klosin A., Casas E., Hidalgo-Carcedo C., Vavouri T., Lehner B. (2017). Transgenerational Transmission of Environmental Information in *C. elegans*. Science.

[194] Skinner M.K. (2015). Environmental Epigenetics and a Unified Theory of the Molecular Aspects of Evolution: A Neo-Lamarckian Concept That Facilitates Neo-Darwinian Evolution. Genome Biol. Evol..

[195] Skinner M.K., Nilsson E.E. (2021). Role of Environmentally Induced Epigenetic Transgenerational Inheritance in Evolutionary Biology: Unified Evolution Theory. Environ. Epigenet..

[196] Morran L.T., Cappy B.J., Anderson J.L., Phillips P.C. (2009). Sexual Partners for the Stressed: Facultative Outcrossing in the Self-Fertilizing Nematode *Caenorhabditis elegans*. Evolution.

[197] Ii R.C.P., Penley M.J., Morran L.T. (2016). The Integral Role of Genetic Variation in the Evolution of Outcrossing in the *Caenorhabditis elegans*-*Serratia marcescens* Host-Parasite System. PLoS ONE.

[198] Leighton D.H.W., Choe A., Wu S.Y., Sternberg P.W. (2014). Communication between Oocytes and Somatic Cells Regulates Volatile Pheromone Production in *Caenorhabditis elegans*. Proc. Natl. Acad. Sci. USA.

[199] Wan X., Zhou Y., Chan C.M., Yang H., Yeung C., Chow K.L. (2019). Chow SRD-1 in AWA Neurons Is the Receptor for Female Volatile Sex Pheromones in *C. elegans* Males. EMBO Rep..

[200] Toker I.A., Lev I., Mor Y., Gurevich Y., Fisher D., Houri-Zeevi L., Antonova O., Doron H., Anava S., Gingold H. (2022). Transgenerational Inheritance of Sexual Attractiveness via Small RNAs Enhances Evolvability in *C. elegans*. Dev. Cell.

[201] Seydoux G. (2018). The P Granules of *C. elegans*: A Genetic Model for the Study of RNA–Protein Condensates. J. Mol. Biol..

[202] Wang J.T., Smith J., Chen B.-C., Schmidt H., Rasoloson D., Paix A., Lambrus B.G., Calidas D., Betzig E., Seydoux G. (2014). Regulation of RNA Granule Dynamics by Phosphorylation of Serine-Rich, Intrinsically Disordered Proteins in *C. elegans*. eLife.

[203] Putnam A., Cassani M., Smith J., Seydoux G. (2019). A Gel Phase Promotes Condensation of Liquid P Granules in *Caenorhabditis elegans* Embryos. Nat. Struct. Mol. Biol..

[204] Campbell A.C., Updike D.L. (2015). CSR-1 and P Granules Suppress Sperm-Specific Transcription in the *C. elegans* Germline. Development.

[205] Sharma U., Rando O.J. (2017). Metabolic Inputs into the Epigenome. Cell Metab..

[206] Aiken C.E., Tarry-Adkins J.L., Ozanne S.E. (2016). Transgenerational Effects of Maternal Diet on Metabolic and Reproductive Ageing. Mamm. Genome.

[207] Panera N., Mandato C., Crudele A., Bertrando S., Vajro P., Alisi A. (2022). Genetics, Epigenetics and Transgenerational Transmission of Obesity in Children. Front. Endocrinol..

[208] Massiera F., Barbry P., Guesnet P., Joly A., Luquet S., Moreilhon-Brest C., Mohsen-Kanson T., Amri E.-Z., Ailhaud G. (2010). A Western-like Fat Diet Is Sufficient to Induce a Gradual Enhancement in Fat Mass over Generations. J. Lipid Res..

[209] Clemente-Suárez V.J., Beltrán-Velasco A.I., Redondo-Flórez L., Martín-Rodríguez A., Tornero-Aguilera J.F. (2023). Global Impacts of Western Diet and Its Effects on Metabolism and Health: A Narrative Review. Nutrients.

[210] Lathigara D., Kaushal D., Wilson R.B. (2023). Molecular Mechanisms of Western Diet-Induced Obesity and Obesity-Related Carcinogenesis—A Narrative Review. Metabolites.

[211] Tadros W., Lipshitz H.D. (2009). The Maternal-to-Zygotic Transition: A Play in Two Acts. Development.

[212] Lee M.T., Bonneau A.R., Giraldez A.J. (2014). Zygotic Genome Activation during the Maternal-to-Zygotic Transition. Annu. Rev. Cell Dev. Biol..

[213] Vastenhouw N.L., Cao W.X., Lipshitz H.D. (2019). The Maternal-to-Zygotic Transition Revisited. Development.

[214] Bazer F.W., Spencer T.E., Wu G., Cudd T.A., Meininger C.J. (2004). Maternal Nutrition and Fetal Development. J. Nutr..

[215] Beauchamp B., Harper M.-E. (2016). In Utero Undernutrition Programs Skeletal and Cardiac Muscle Metabolism. Front. Physiol..

[216] Gómez-Gallego C., García-Mantrana I., Martínez-Costa C., Salminen S., Isolauri E., Collado M.C. (2019). The Microbiota and Malnutrition: Impact of Nutritional Status during Early Life. Annu. Rev. Nutr..

[217] Shan S., Xu F., Hirschfeld M., Brenig B. (2021). Sperm Lipid Markers of Male Fertility in Mammals. Int. J. Mol. Sci..

[218] Mostafa S., Nader N., Machaca K. (2022). Lipid Signaling During Gamete Maturation. Front. Cell Dev. Biol..

[219] L’Hernault S.W. (2006). Spermatogenesis. WormBook: The Online Review of C. elegans Biology [Internet].

[220] Cheng C.Y., Mruk D.D. (2010). The Biology of Spermatogenesis: The Past, Present and Future. Philos. Trans. R. Soc. B Biol. Sci..

[221] Reilly J.N., McLaughlin E.A., Stanger S.J., Anderson A.L., Hutcheon K., Church K., Mihalas B.P., Tyagi S., Holt J.E., Eamens A.L. (2016). Characterisation of Mouse Epididymosomes Reveals a Complex Profile of MicroRNAs and a Potential Mechanism for Modification of the Sperm Epigenome. Sci. Rep..

[222] Kappil M., Wright R.O., Sanders A.P. (2016). Sanders Developmental Origins of Common Disease: Epigenetic Contributions to Obesity. Annu. Rev. Genom. Hum. Genet..

[223] Dupont C., Kappeler L., Saget S., Grandjean V., Lévy R. (2019). Role of MiRNA in the Transmission of Metabolic Diseases Associated With Paternal Diet-Induced Obesity. Front. Genet..

[224] Kaspar D., Hastreiter S., Irmler M., Hrabé de Angelis M., Beckers J. (2020). Nutrition and Its Role in Epigenetic Inheritance of Obesity and Diabetes across Generations. Mamm. Genome.

[225] King S.E., Skinner M.K. (2020). Epigenetic Transgenerational Inheritance of Obesity Susceptibility. Trends Endocrinol. Metab..

[226] Ng S.-F., Lin R.C.Y., Laybutt D.R., Barres R., Owens J.A., Morris M.J. (2010). Chronic High-Fat Diet in Fathers Programs β 2-Cell Dysfunction in Female Rat Offspring. Nature.

[227] Huypens P., Sass S., Wu M., Dyckhoff D., Tschöp M., Theis F., Marschall S., de Angelis M.H., Beckers J. (2016). Epigenetic Germline Inheritance of Diet-Induced Obesity and Insulin Resistance. Nat. Genet..

[228] de Castro Barbosa T., Ingerslev L.R., Alm P.S., Versteyhe S., Massart J., Rasmussen M., Donkin I., Sjögren R., Mudry J.M., Vetterli L. (2015). High-Fat Diet Reprograms the Epigenome of Rat Spermatozoa and Transgenerationally Affects Metabolism of the Offspring. Mol. Metab..

[229] Zhang Y., Zhang X., Shi J., Tuorto F., Li X., Liu Y., Liebers R., Zhang L., Qu Y., Qian J. (2018). Dnmt2 Mediates Intergenerational Transmission of Paternally Acquired Metabolic Disorders through Sperm Small Non-Coding RNAs. Nat. Cell Biol..

[230] Cropley J.E., Eaton S.A., Aiken A., Young P.E., Giannoulatou E., Ho J.W.K., Buckland M.E., Keam S.P., Hutvagner G., Humphreys D.T. (2016). Male-Lineage Transmission of an Acquired Metabolic Phenotype Induced by Grand-Paternal Obesity. Mol. Metab..

[231] Carone B.R., Fauquier L., Habib N., Shea J.M., Hart C.E., Li R., Bock C., Li C., Gu H., Zamore P.D. (2010). Paternally Induced Transgenerational Environmental Reprogramming of Metabolic Gene Expression in Mammals. Cell.

[232] Billi A.C., Freeberg M.A., Kim J.K. (2012). PiRNAs and SiRNAs Collaborate in *Caenorhabditis elegans* Genome Defense. Genome Biol..

[233] Schlee M., Hartmann G. (2016). Discriminating Self from Non-Self in Nucleic Acid Sensing. Nat. Rev. Immunol..

[234] Cornec A., Poirier E.Z. (2023). Interplay between RNA Interference and Transposable Elements in Mammals. Front. Immunol..

[235] Palominos M.F., Verdugo L., Gabaldon C., Pollak B., Ortíz-Severín J., Varas M.A., Chávez F.P., Calixto A. (2017). Transgenerational Diapause as an Avoidance Strategy against Bacterial Pathogens in *Caenorhabditis elegans*. mBio.

[236] Burton N.O., Riccio C., Dallaire A., Price J., Jenkins B., Koulman A., Miska E.A. (2020). Cysteine Synthases CYSL-1 and CYSL-2 Mediate *C. elegans* Heritable Adaptation to *P. vranovensis* Infection. Nat. Commun..

[237] Pereira A.G., Gracida X., Kagias K., Zhang Y. (2020). *C. elegans* Aversive Olfactory Learning Generates Diverse Intergenerational Effects. J. Neurogenet..

[238] Houri-Ze’evi L., Korem Y., Sheftel H., Faigenbloom L., Toker I.A., Dagan Y., Awad L., Degani L., Alon U., Rechavi O. (2016). A Tunable Mechanism Determines the Duration of the Transgenerational Small RNA Inheritance in *C. elegans*. Cell.

[239] Houri-Zeevi L., Korem Kohanim Y., Antonova O., Rechavi O. (2020). Three Rules Explain Transgenerational Small RNA Inheritance in *C. elegans*. Cell.

[240] Houri-Zeevi L., Teichman G., Gingold H., Rechavi O. (2021). Stress Resets Ancestral Heritable Small RNA Responses. eLife.

[241] Meisel J.D., Panda O., Mahanti P., Schroeder F.C., Kim D.H. (2014). Chemosensation of Bacterial Secondary Metabolites Modulates Neuroendocrine Signaling and Behavior of *C. elegans*. Cell.

[242] Kudlow B.A., Zhang L., Han M. (2012). Systematic Analysis of Tissue-Restricted MiRISCs Reveals a Broad Role for MicroRNAs in Suppressing Basal Activity of the *C. elegans* Pathogen Response. Mol. Cell.

[243] Liu F., He C.-X., Luo L.-J., Zou Q.-L., Zhao Y.-X., Saini R., Han S.-F., Knölker H.-J., Wang L.-S., Ge B.-X. (2013). Nuclear Hormone Receptor Regulation of MicroRNAs Controls Innate Immune Responses in *C. elegans*. PLoS Pathog..

[244] Dai L.-L., Gao J.-X., Zou C.-G., Ma Y.-C., Zhang K.-Q. (2015). Mir-233 Modulates the Unfolded Protein Response in *C. elegans* during Pseudomonas Aeruginosa Infection. PLoS Pathog..

[245] Ren Z., Ambros V.R. (2015). *Caenorhabditis elegans* MicroRNAs of the Let-7 Family Act in Innate Immune Response Circuits and Confer Robust Developmental Timing against Pathogen Stress. Proc. Natl. Acad. Sci. USA.

[246] Ma Y.-C., Zhang L., Dai L.-L., Khan R.U., Zou C.-G. (2017). Mir-67 Regulates P. Aeruginosa Avoidance Behavior in *C. elegans*. Biochem. Biophys. Res. Commun..

[247] Kuvbachieva A., Bestel A.-M., Tissir F., Maloum I., Guimiot F., Ramoz N., Bourgeois F., Moalic J.-M., Goffinet A.M., Simonneau M. (2004). Identification of a Novel Brain-Specific and Reelin-Regulated Gene That Encodes a Protein Colocalized with Synapsin. Eur. J. Neurosci..

[248] Arellano-Carbajal F., Briseño-Roa L., Couto A., Cheung B.H.H., Labouesse M., Bono M. (2011). de Macoilin, a Conserved Nervous System–Specific ER Membrane Protein That Regulates Neuronal Excitability. PLoS Genet..

[249] Miyara A., Ohta A., Okochi Y., Tsukada Y., Kuhara A., Mori I. (2011). Novel and Conserved Protein Macoilin Is Required for Diverse Neuronal Functions in *Caenorhabditis elegans*. PLoS Genet..

[250] Neal S.J., Park J., DiTirro D., Yoon J., Shibuya M., Choi W., Schroeder F.C., Butcher R.A., Kim K., Sengupta P. (2016). A Forward Genetic Screen for Molecules Involved in Pheromone-Induced Dauer Formation in *Caenorhabditis elegans*. G3 Genes Genomes Genet..

[251] Moore R.S., Kaletsky R., Lesnik C., Cota V., Blackman E., Parsons L.R., Gitai Z., Murphy C.T. (2021). The Role of the Cer1 Transposon in Horizontal Transfer of Transgenerational Memory. Cell.

[252] Dennis S., Sheth U., Feldman J.L., English K.A., Priess J.R. (2012). *C. elegans* Germ Cells Show Temperature and Age-Dependent Expression of Cer1, a Gypsy/Ty3-Related Retrotransposon. PLoS Pathog..

[253] Feinberg E.H., Hunter C.P. (2003). Transport of DsRNA into Cells by the Transmembrane Protein SID-1. Science.

[254] Shih J.D., Fitzgerald M.C., Sutherlin M., Hunter C.P. (2009). The SID-1 Double-Stranded RNA Transporter Is Not Selective for DsRNA Length. RNA.

[255] Minkina O., Hunter C.P. (2018). Intergenerational Transmission of Gene Regulatory Information in *Caenorhabditis elegans*. Trends Genet..

[256] Marré J., Traver E.C., Jose A.M. (2016). Extracellular RNA Is Transported from One Generation to the next in *Caenorhabditis elegans*. Proc. Natl. Acad. Sci. USA.

[257] Wang E., Hunter C.P. (2017). SID-1 Functions in Multiple Roles to Support Parental RNAi in *Caenorhabditis elegans*. Genetics.

[258] Ren R., Xu X., Lin T., Weng S., Liang H., Huang M., Dong C., Luo Y., He J. (2011). Cloning, Characterization, and Biological Function Analysis of the SidT2 Gene from Siniperca Chuatsi. Dev. Comp. Immunol..

[259] Elhassan M.O., Christie J., Duxbury M.S. (2012). Homo Sapiens Systemic RNA Interference-Defective-1 Transmembrane Family Member 1 (SIDT1) Protein Mediates Contact-Dependent Small RNA Transfer and MicroRNA-21-Driven Chemoresistance. J. Biol. Chem..

[260] Pratt A.J., Rambo R.P., Lau P.-W., MacRae I.J. (2012). Preparation and Characterization of the Extracellular Domain of Human Sid-1. PLoS ONE.

[261] Rouhana L., Weiss J.A., Forsthoefel D.J., Lee H., King R.S., Inoue T., Shibata N., Agata K., Newmark P.A. (2013). RNA Interference by Feeding in Vitro–Synthesized Double-Stranded RNA to Planarians: Methodology and Dynamics. Dev. Dyn..

[262] Li W., Koutmou K.S., Leahy D.J., Li M. (2015). Systemic RNA Interference Deficiency-1 (SID-1) Extracellular Domain Selectively Binds Long Double-Stranded RNA and Is Required for RNA Transport by SID-1. J. Biol. Chem..

[263] Kwak J.E., Wickens M. (2007). A Family of Poly(U) Polymerases. RNA.

[264] Spracklin G., Fields B., Wan G., Becker D., Wallig A., Shukla A., Kennedy S. (2017). The RNAi Inheritance Machinery of *Caenorhabditis elegans*. Genetics.

[265] Sapetschnig A., Sarkies P., Lehrbach N.J., Miska E.A. (2015). Tertiary SiRNAs Mediate Paramutation in *C. elegans*. PLoS Genet..

[266] Gu S.G., Pak J., Guang S., Maniar J.M., Kennedy S., Fire A. (2012). A Transgenerational Impact of SiRNA on Chromatin: SiRNA Amplification in *Caenorhabditis elegans* Generates a Homology-Targeted Footprint of H3K9 Methylated Nucleosomes. Nat. Genet..

[267] Shukla A., Yan J., Pagano D.J., Dodson A.E., Fei Y., Gorham J., Seidman J.G., Wickens M., Kennedy S. (2020). Poly(UG)-Tailed RNAs in Genome Protection and Epigenetic Inheritance. Nature.

[268] Preston M.A., Porter D.F., Chen F., Buter N., Lapointe C.P., Keles S., Kimble J., Wickens M. (2019). Unbiased Screen of RNA Tailing Activities Reveals a Poly(UG) Polymerase. Nat. Methods.

[269] Collins J., Saari B., Anderson P. (1987). Activation of a Transposable Element in the Germ Line but Not the Soma of *Caenorhabditis elegans*. Nature.

[270] Ketting R.F., Haverkamp T.H.A., van Luenen H.G.A.M., Plasterk R.H.A. (1999). Mut-7 of *C. elegans*, Required for Transposon Silencing and RNA Interference, Is a Homolog of Werner Syndrome Helicase and RNaseD. Cell.

[271] Tabara H., Sarkissian M., Kelly W.G., Fleenor J., Grishok A., Timmons L., Fire A., Mello C.C. (1999). The Rde-1 Gene, RNA Interference, and Transposon Silencing in *C. elegans*. Cell.

[272] Chen C.-C.G., Simard M.J., Tabara H., Brownell D.R., McCollough J.A., Mello C.C. (2005). A Member of the Polymerase β Nucleotidyltransferase Superfamily Is Required for RNA Interference in *C. elegans*. Curr. Biol..

[273] Roschdi S., Yan J., Nomura Y., Escobar C.A., Petersen R.J., Bingman C.A., Tonelli M., Vivek R., Montemayor E.J., Wickens M. (2022). An Atypical RNA Quadruplex Marks RNAs as Vectors for Gene Silencing. Nat. Struct. Mol. Biol..

[274] Gent J.I., Lamm A.T., Pavelec D.M., Maniar J.M., Parameswaran P., Tao L., Kennedy S., Fire A.Z. (2010). Distinct Phases of SiRNA Synthesis in an Endogenous RNAi Pathway in *C. elegans* Soma. Mol. Cell.

[275] Varshney D., Spiegel J., Zyner K., Tannahill D., Balasubramanian S. (2020). The Regulation and Functions of DNA and RNA G-Quadruplexes. Nat. Rev. Mol. Cell Biol..

[276] Kharel P., Fay M., Manasova E.V., Anderson P.J., Kurkin A.V., Guo J.U., Ivanov P. (2023). Stress Promotes RNA G-Quadruplex Folding in Human Cells. Nat. Commun..

[277] Pitt J.N., Schisa J.A., Priess J.R. (2000). P Granules in the Germ Cells of *Caenorhabditis elegans* Adults Are Associated with Clusters of Nuclear Pores and Contain RNA. Dev. Biol..

[278] Lee C.-Y.S., Putnam A., Lu T., He S., Ouyang J.P.T., Seydoux G. (2020). Recruitment of MRNAs to P Granules by Condensation with Intrinsically-Disordered Proteins. eLife.

[279] Shukla A., Perales R., Kennedy S. (2021). PiRNAs Coordinate Poly(UG) Tailing to Prevent Aberrant and Perpetual Gene Silencing. Curr. Biol..

[280] Phillips C.M., Updike D.L. (2022). Germ Granules and Gene Regulation in the *Caenorhabditis elegans* Germline. Genetics.

[281] Burton N.O., Willis A., Fisher K., Braukmann F., Price J., Stevens L., Baugh L.R., Reinke A., Miska E.A. (2021). Intergenerational Adaptations to Stress Are Evolutionarily Conserved, Stress-Specific, and Have Deleterious Trade-Offs. eLife.

[282] Liu H., Zhao Y., Hua X., Wang D. (2022). Induction of Transgenerational Toxicity Is Associated with the Activated Germline Insulin Signals in Nematodes Exposed to Nanoplastic at Predicted Environmental Concentrations. Ecotoxicol. Environ. Saf..

[283] Jablonka E., Raz G. (2009). Transgenerational Epigenetic Inheritance: Prevalence, Mechanisms, and Implications for the Study of Heredity and Evolution. Q. Rev. Biol..

[284] Wang Y., Liu H., Sun Z. (2017). Lamarck Rises from His Grave: Parental Environment-Induced Epigenetic Inheritance in Model Organisms and Humans. Biol. Rev..

[285] Horsthemke B. (2018). A Critical View on Transgenerational Epigenetic Inheritance in Humans. Nat. Commun..

[286] Roth O., Beemelmanns A., Barribeau S.M., Sadd B.M. (2018). Recent Advances in Vertebrate and Invertebrate Transgenerational Immunity in the Light of Ecology and Evolution. Heredity.

[287] Skvortsova K., Iovino N., Bogdanović O. (2018). Functions and Mechanisms of Epigenetic Inheritance in Animals. Nat. Rev. Mol. Cell Biol..

[288] Senaldi L., Smith-Raska M. (2020). Evidence for Germline Non-Genetic Inheritance of Human Phenotypes and Diseases. Clin. Epigenetics.

[289] Alcazar R.M., Lin R., Fire A.Z. (2008). Transmission Dynamics of Heritable Silencing Induced by Double-Stranded RNA in *Caenorhabditis elegans*. Genetics.

[290] Vastenhouw N.L., Brunschwig K., Okihara K.L., Müller F., Tijsterman M., Plasterk R.H.A. (2006). Long-Term Gene Silencing by RNAi. Nature.

[291] Kalinava N., Ni J.Z., Peterman K., Chen E., Gu S.G. (2017). Decoupling the Downstream Effects of Germline Nuclear RNAi Reveals That H3K9me3 Is Dispensable for Heritable RNAi and the Maintenance of Endogenous SiRNA-Mediated Transcriptional Silencing in *Caenorhabditis elegans*. Epigenet. Chromatin.

[292] Kalinava N., Ni J.Z., Gajic Z., Kim M., Ushakov H., Gu S.G. (2018). *C. elegans* Heterochromatin Factor SET-32 Plays an Essential Role in Transgenerational Establishment of Nuclear RNAi-Mediated Epigenetic Silencing. Cell Rep..

[293] Woodhouse R.M., Buchmann G., Hoe M., Harney D.J., Low J.K.K., Larance M., Boag P.R., Ashe A. (2018). Chromatin Modifiers SET-25 and SET-32 Are Required for Establishment but Not Long-Term Maintenance of Transgenerational Epigenetic Inheritance. Cell Rep..

[294] Andersen E.C., Horvitz H.R. (2007). Two *C. elegans* Histone Methyltransferases Repress Lin-3EGF Transcription to Inhibit Vulval Development. Development.

[295] Greer E.L., Beese-Sims S.E., Brookes E., Spadafora R., Zhu Y., Rothbart S.B., Aristizábal-Corrales D., Chen S., Badeaux A.I., Jin Q. (2014). A Histone Methylation Network Regulates Transgenerational Epigenetic Memory in *C. elegans*. Cell Rep..

[296] Lev I., Seroussi U., Gingold H., Bril R., Anava S., Rechavi O. (2017). MET-2-Dependent H3K9 Methylation Suppresses Transgenerational Small RNA Inheritance. Curr. Biol..

[297] Kerr S.C., Ruppersburg C.C., Francis J.W., Katz D.J. (2014). SPR-5 and MET-2 Function Cooperatively to Reestablish an Epigenetic Ground State during Passage through the Germ Line. Proc. Natl. Acad. Sci. USA.

[298] Perales R., Pagano D., Wan G., Fields B.D., Saltzman A.L., Kennedy S.G. (2018). Transgenerational Epigenetic Inheritance Is Negatively Regulated by the HERI-1 Chromodomain Protein. Genetics.

[299] Zhuang J.J., Hunter C.P. (2012). The Influence of Competition Among *C. elegans* Small RNA Pathways on Development. Genes.

[300] Brunquell J., Morris S., Lu Y., Cheng F., Westerheide S.D. (2016). The Genome-Wide Role of HSF-1 in the Regulation of Gene Expression in *Caenorhabditis elegans*. BMC Genom..

[301] Joutsen J., Sistonen L. (2019). Tailoring of Proteostasis Networks with Heat Shock Factors. Cold Spring Harb. Perspect. Biol..

[302] Brunquell J., Snyder A., Cheng F., Westerheide S.D. (2017). HSF-1 Is a Regulator of MiRNA Expression in *Caenorhabditis elegans*. PLoS ONE.

[303] Schreiner W.P., Pagliuso D.C., Garrigues J.M., Chen J.S., Aalto A.P., Pasquinelli A.E. (2019). Remodeling of the *Caenorhabditis elegans* Non-Coding RNA Transcriptome by Heat Shock. Nucleic Acids Res..

[304] Zheng X., Beyzavi A., Krakowiak J., Patel N., Khalil A.S., Pincus D. (2018). Hsf1 Phosphorylation Generates Cell-to-Cell Variation in Hsp90 Levels and Promotes Phenotypic Plasticity. Cell Rep..

[305] Andrusiak M.G., Jin Y. (2016). Context Specificity of Stress-Activated Mitogen-Activated Protein (MAP) Kinase Signaling: The Story as Told by *Caenorhabditis elegans*. J. Biol. Chem..

[306] Blackwell T.K., Steinbaugh M.J., Hourihan J.M., Ewald C.Y., Isik M. (2015). SKN-1/Nrf, Stress Responses, and Aging in *Caenorhabditis elegans*. Free Radic. Biol. Med..

[307] Ow M.C., Nichitean A.M., Hall S.E. (2021). Somatic Aging Pathways Regulate Reproductive Plasticity in *Caenorhabditis elegans*. eLife.

[308] Updike D.L., Knutson A.K., Egelhofer T.A., Campbell A.C., Strome S. (2014). Germ-Granule Components Prevent Somatic Development in the *C. elegans* Germline. Curr. Biol..

[309] Rochester J.D., Min H., Gajjar G.A., Sharp C.S., Maki N.J., Rollins J.A., Keiper B.D., Graber J.H., Updike D.L. (2022). GLH-1/Vasa Represses Neuropeptide Expression and Drives Spermiogenesis in the *C. elegans* Germline. Dev. Biol..

[310] Chen W., Brown J.S., He T., Wu W.-S., Tu S., Weng Z., Zhang D., Lee H.-C. (2022). GLH/VASA Helicases Promote Germ Granule Formation to Ensure the Fidelity of PiRNA-Mediated Transcriptome Surveillance. Nat. Commun..

[311] Spike C.A., Bader J., Reinke V., Strome S. (2008). DEPS-1 Promotes P-Granule Assembly and RNA Interference in *C. elegans* Germ Cells. Development.

[312] Suen K.M., Braukmann F., Butler R., Bensaddek D., Akay A., Lin C.-C., Milonaitytė D., Doshi N., Sapetschnig A., Lamond A. (2020). DEPS-1 Is Required for PiRNA-Dependent Silencing and PIWI Condensate Organisation in *Caenorhabditis elegans*. Nat. Commun..

[313] Nguyen D.A.H., Phillips C.M. (2021). Arginine Methylation Promotes SiRNA-Binding Specificity for a Spermatogenesis-Specific Isoform of the Argonaute Protein CSR-1. Nat. Commun..

